# Multiscale (microscopic to remote sensing) preliminary exploration of auriferous-uraniferous marbles: A case study from the Egyptian Nubian Shield

**DOI:** 10.1038/s41598-023-36388-7

**Published:** 2023-06-06

**Authors:** Ali Shebl, Mohamed Hamdy

**Affiliations:** 1 Department of Mineralogy and Geology, University of Debrecen, Debrecen, 4032 Hungary; 2 Geology Department, Tanta University, Tanta, 31527 Egypt

**Keywords:** Mineralogy, Geology

## Abstract

Since their recent first record within the Egyptian Nubian Shield, auriferous and uraniferous marbles (Au = 0.98–2.76 g/t; U = 133–640 g/t) have rarely been addressed, despite not only their probable economic importance but also the fact that it is a new genetic style of gold and uranium mineralization in the Nubian Shield rocks. This is mainly attributed to the inadequate localization of these marbles within harsh terrains, as well as the cost and time spent with conventional fieldwork for their identification compared to the main lithological components of the Nubian Shield. On the contrary, remote sensing and machine learning techniques save time and effort while introducing reliable feature identification with reasonable accuracy. Consequently, the current research is an attempt to apply the well-known machine learning algorithm (Support vector Machine—SVM) over Sentinel 2 remote sensing data (with a spatial resolution of up to 10 m) to delineate the distribution of auriferous-uraniferous marbles in the Barramiya-Daghbagh district (Eastern Desert of Egypt), as a case study from the Nubian Shield. Towards better results, marbles were accurately distinguished utilizing ALOS PRISM (2.5 m) pan-sharpened Sentinel 2 data and well-known exposures during fieldwork. With an overall accuracy of more than 90%, a thematic map for auriferous-uraniferous marbles and the major rock units in the Barramiya-Daghbagh district was produced. Marbles are spatially related to ophiolitic serpentinite rocks, as consistent with their genesis within the Neoproterozoic oceanic lithosphere. Field and petrographic investigations have confirmed the newly detected Au and U-bearing zones (impure calcitic to impure dolomitic marbles in Wadi Al Barramiya and Wadi Daghbagh areas and impure calcitic marble in Gebel El-Rukham area). Additionally, X-ray diffraction (XRD), back-scattered electron images (BSEIs), and Energy Dispersive X-ray spectroscopy (EDX) results were integrated to verify our remote sensing results and petrographic investigations. Different times of mineralization are indicated, ranging from syn-metamorphism (gold in Wadi Al Barramiya and Gebel El-Rukham) to post-metamorphism (gold in Wadi Daghbagh and uranium in all locations). Based on the application of geological, mineralogical, machine learning and remote sensing results for the construction of a preliminary exploration model of the auriferous-uraniferous marble in the Egyptian Nubian Shield, we recommend a detailed exploration of Au and U-bearing zones in Barramiya-Dghbagh district and applying the adopted approach to other districts of similar geological environments.

## Introduction

Marble in the proper sense (a coarse-grained metamorphosed calcitic or dolomitic rock of any origin) is known to occur in many localities within the Arabian Nubian Shield (ANS) rocks^1^. Their main occurrences in the shield rocks in the Eastern Desert of Egypt are at Wadi Dib^1^, Wadi Barramiya, Wadi Dghbagh, Gebel El-Rukham off Wadi El-Miyah^2^, Bir Safsaf-Aswan uplift^3^, Wadi Allaqi^4^, and Sol Hamid^5^. In addition, foraminiferal graphitic marbles from the Phanerozoic rocks are detected. They have been found in the Gebel El Hisinat and Wadi Heimur areas, where arenaceous foraminifera of Pennsylvanian and Mississippian ages have been described^6,7^.

Egypt has conspicuously exposed gold and uranium resources that formed at various stages of its geological evolution. Gold deposits occur either as stratabound formed due to exhalative hydrothermal processes during the last stages of sub-marine volcanic activity in island arcs^8,9^, vein-type^10,11^, disseminated –type in altered rocks^12^ or as placers^13^. The main uranium occurrences are found in shear zones in the Pan-African late orogenic granite and related rocks^14^, alkaline dykes and sills^15^, Phanerozoic sedimentary rocks^16,17^ and beach placers of the black sand^18^. However, due to their considerable chemical favorability for infiltration by hydrothermal fluids, carbonate rocks are a well-known host for various types of hydrothermal alteration and metasomatism-related mineralization including gold^19–21^, uranium^22–25^, and rare earth elements^26,27^. Furthermore, during and after the metamorphism of carbonate rocks, the movement of mineralizing solutions can transfer elements from the surrounding rocks into the produced marble^28–32^. Therefore, marbles are considered a potential host for many ores and are documented for gold and uranium settling down within ANS rocks^2^. Despite the frequent and thorough investigations for carbonate alteration-related mineralization, their small size and lack of recording on large-scale geological maps^12,20,33,34^, hinder marbles investigations as a host for economic mineral deposits within the ANS. In addition to the critical need for exploration-based economic evaluation of the ANS's recorded Au and U-bearing marbles (to decipher their potential as a new gold and uranium geological trap), research into their origin can provide new insights into the conventional tectonic model of ANS^2,35^.

The globally increasing demand for Au and U, and the advances in metallurgical technologies for ore exploration and exploitation strongly revive mineralogical studies in brownfields and small-scale widely distributed economic deposits. Moreover and with the advent of higher spatial resolution remote sensing datasets, accurate lithological mapping could be achieved^36–43^ even for small-scale rock bodies. Supplementing remote sensing data with machine learning algorithms (MLAs) helps in predicting a certain class (rock type) based on its labeled data in what is known as supervised classification^39,44–49^. In this way, the small size distribution problem of auriferous-uraniferous marbles could be solved besides highlighting new occurrences and introducing a thematic mapping of them. Towards this end, SVM was picked out as MLA to implement this task over Sentinel 2 data due to their well-reported results in similar applications^50–56^. Through a comprehensive approach, this research combines high-resolution remote sensing data (up to 2.5 m) with machine learning, extensive fieldwork, and in-depth mineralogical investigations involving XRD, EDX, and BSEIs. The culmination of these efforts is the creation of a detailed thematic map specifically focused on identifying auriferous-uraniferous marbles within the Barramiya-Daghbagh district, located in the Egyptian Nubian Shield. This study seeks to assess whether this exploration model is sufficient to warrant additional, potentially costly, exploration for auriferous-uraniferous marbles throughout the entire ANS and other districts with similar geological settings.

## Regional geology

**Figure 1:**
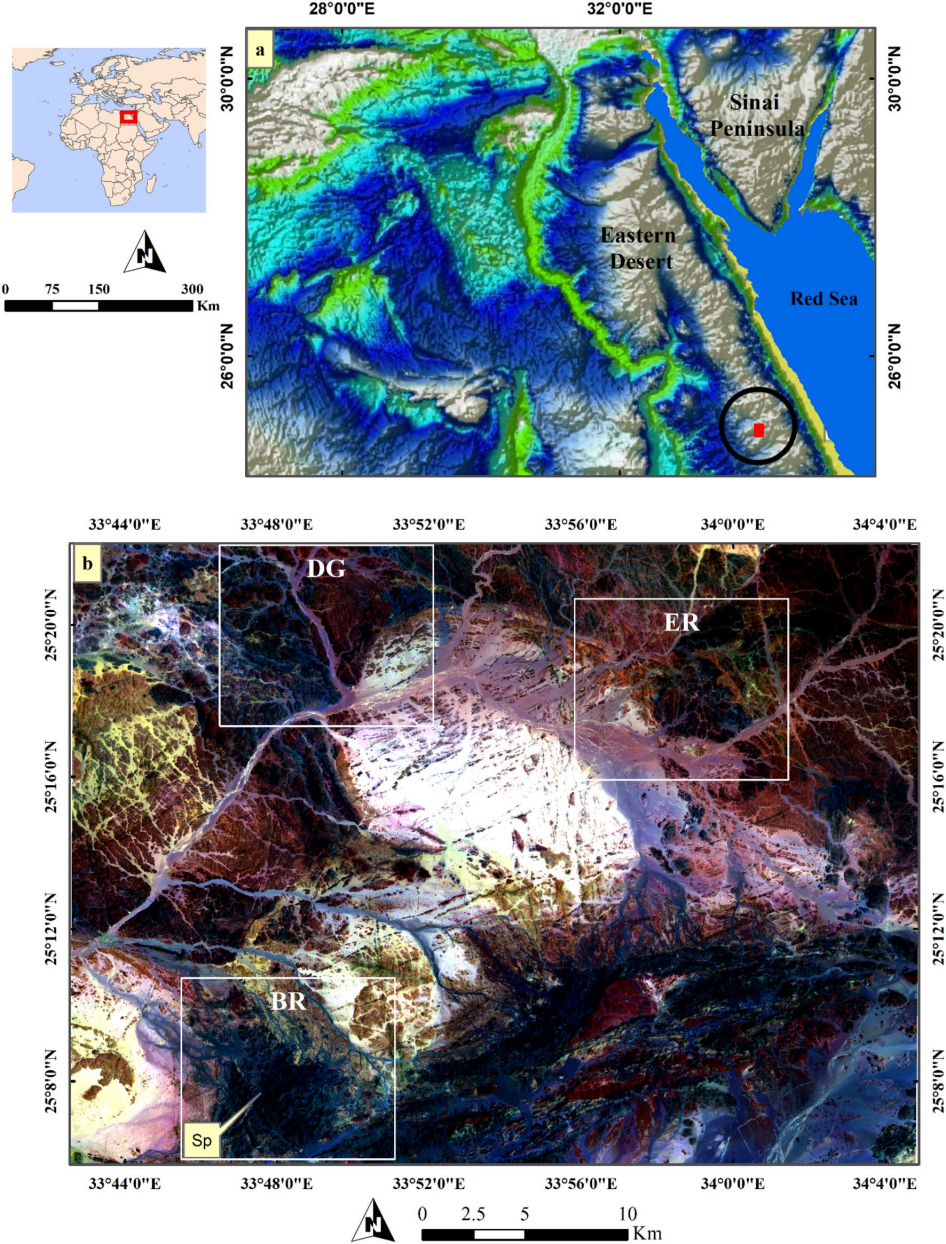
(**a**) Location map of the study area and (**b**) Sentinel 2 FCC 12–6-2 in RGB respectively showing serpentinite rocks in black color. BR: Barramiya district , ER: El-Rukham district, DG: Daghbagh district, Sp: serpentinite and its related rocks including Au-U-bearing marbles. (Sentinel 2A image was downloaded through the European Space Agency (ESA) platform. The figure was created by SmartSketch v. 4.0 software; https://smartsketch.software.informer.com/4.0/ and ENVI v. 5.6.2. software; (https://www.l3harrisgeospatial.com/Software-Technology/ENVI).

The Barramiya-Daghbagh district is located in the Eastern Desert of Egypt, as a part of the ANS. In Egypt, the Precambrian basement in the Eastern Desert and the Sinai Peninsula constitutes the northern part of the Nubian shield in the ANS (Fig. 1a). The ANS, forming one of the largest exposures of Neoproterozoic juvenile crust on Earth^57^, extends from Egypt through Sudan and Eritrea to Ethiopia on the western side of the Red Sea rift, and from Palestine and Jordan through Saudi Arabia to Yemen on the eastern side of the Red Sea. It was developed by accretion of island arcs to the Gondwana continental margins by the closure of the Mozambique Ocean during the East-African orogen, followed by crustal extension that was accompanied by the intrusion of large amounts of granitoid magmas (750–540 Ma)^58^ and generation of post-amalgamation depositional basins^59,60^ in which volcano-sedimentary rocks abound (< 650 Ma^61^). Arc amalgamation began about 780 Ma and continued to about 620 Ma^62^ and the overall shield assembly terminated at about 560 Ma, by which time the ANS had been accreted to the Saharan Metacraton^4^. Subduction was active while the process of obduction was operative along the thrust planes^57^.

The Precambrian basement in the Egyptian Nubian shield are products of magmatic, sedimentary and metamorphic processes of a complex Proterozoic orogenic evolution following terrane collision and accretion onto a pre-Pan-African continent to the west of the Nile^63^. The rocks of the collision area may have been subjected to regional metamorphism between 650 and 620 Ma ago^64^. They were incorporated by thrusting during accretion and by a left-lateral transcurrent movement along the Najd and other NW-striking shear zones, particularly in the central part of the Eastern Desert of Egypt^65,66^.

## Geology of Barramiya-Daghbagh district

**Figure 2:**
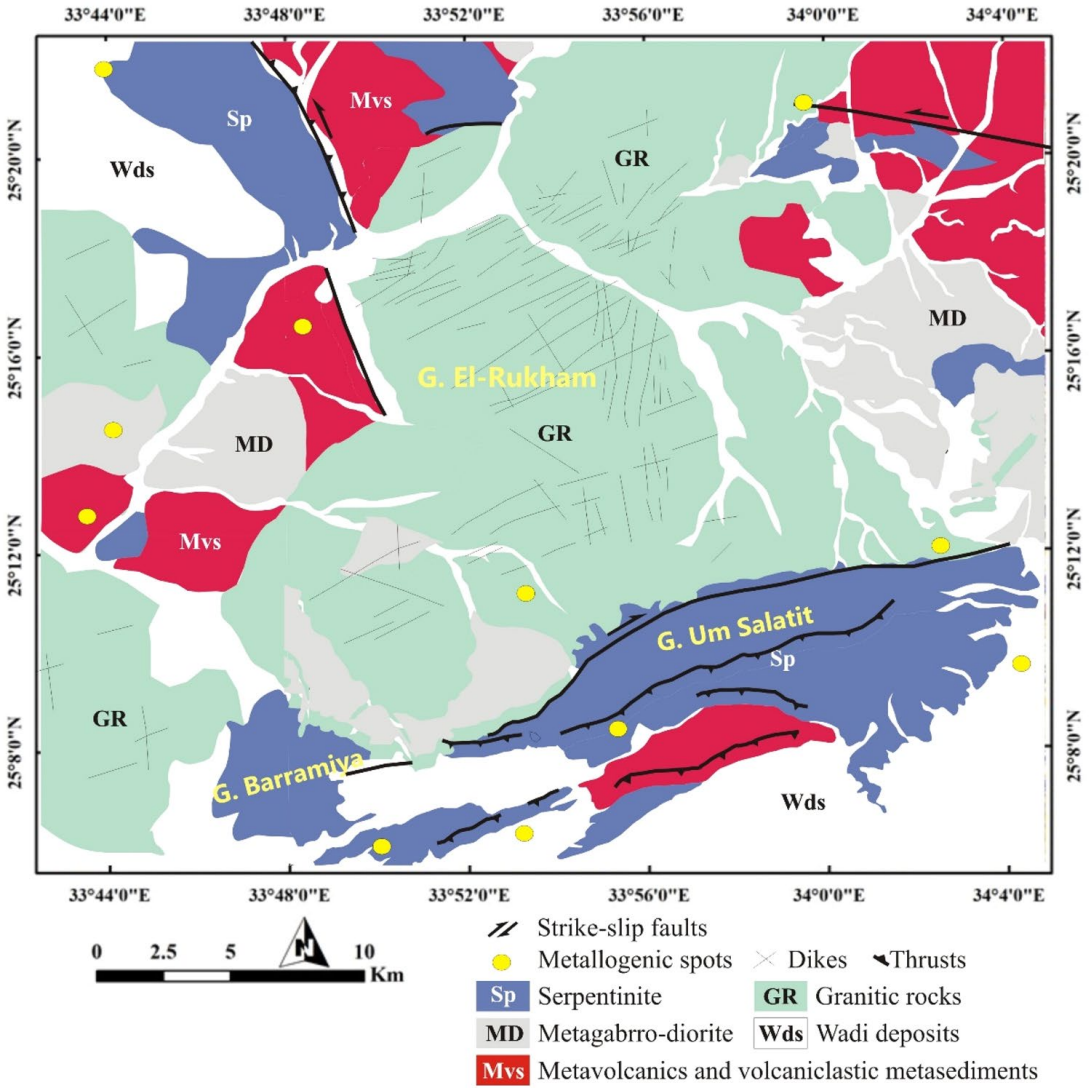
Geological map of Barramiya- Daghbagh district modified after Hagag and Abdelnasser^75^, Shebl, Kusky, and Csámer^76^; and Zoheir et al.^77^. (Created by SmartSketch v. 4.0 software; https://smartsketch.software.informer.com/4.0/).

The Barramiya-Daghbagh district is located in the southern part of the Central Eastern Desert of Egypt between latitudes 25°06″ to 25°22″ N and longitudes 33°42″ to 34°05″ E (Fig. 1b). The Barramiya-Daghbagh district is made up of metamorphosed dismembered ophiolitic serpentinized ultramafics, gabbros, and volcanics, intrusive metagabbro to metadiorite, island-arc metavolcanics-metasediments, foliated granodiorite and alkali feldspar granite (Fig. 2). Ophiolitic rocks are oceanic lithosphere remnants formed by seafloor spreading above an active subduction zone^67,68^. They are remarkably abundant in the Barramiya-Daghbagh district. Serpentinites mostly occur as massive rocks of elongated ranges defining folded tabular bodies or sheets^69^, and are prevailed by ENE–WSW-directed thrusts marking the Mubarak–Barramiya shear zone of the Eastern Desert of Egypt^70^. Sheared and talcified-carbonatized serpentinites are encountered in association with the other mélange rocks^71^. They have a clear schistose nature in places, giving rise to complete talc-carbonate schist.

Volcanogenic metasediments conformably overly the serpentinite rocks across most of the district. The volcanogenic metasediments alternating with metavolcanics are the largest outcropping units, particularly in the northern part of the Barramiya-Daghbagh district. They are rather heterogeneous, consisting mainly of pelitic and calcareous schists with subordinate quartzofeldspathic schists. The schists are commonly bedded and highly foliated. Mature metamorphosed sediments of sandstones and carbonates are less common. However, metamorphosed arc volcanic (andesite, basaltic meta-andesite, interbedded with dacitic tuffs) slices sometimes occur in tectonic contact with metasediments. Mélange occurs dominantly in association with the metasediments and metavolcanics.

The serpentinites, metasediments, and metavolcanics are intruded by metagabbro-diorite rock complexes, which sometimes contain xenoliths and rafts of these rocks. The metagabbro-diorite complexes occur mainly to the east of G. El-Rukham at W. Al Miyah (Fig. 2). The rocks of the complex represent a part of the voluminous plutonic arc-related association^72–74^. They were mildly deformed and metamorphosed to greenschist-amphibolite facies^75,76^. Moreover, the alkali-feldspar granite shows apophyses intruded into the metagabbro-diorite. The basement rocks of the Barramiya-Daghbagh district are commonly traversed by a number of ENE-WSW trending mafic and felsic dykes.

## Materials and methods

### Remote sensing data

To achieve the aim of the current research, a cloud-free Sentinel 2A image was downloaded through the European Space Agency (ESA) platform. Spectral and spatial characteristics of Sentinel 2 data are summarized in Table 1. Sentinel 2 data was reprojected to the datum of WGS-84 UTM zone 36 N. Sen2Cor tool was used for preprocessing (atmospheric correction) Sentinel-2 data to provide corrected bottom-of-atmosphere (BOA) reflectance values from top-of-atmosphere (TOA) Level 1C data (the current scene; (S2A_MSIL1C_20200505T081611_N0209_R121_T36RWN_20200505T095132)). This process was performed by installing the Sen2Cor tool and providing L1C data by some codes using the command prompt.. Herein, Panchromatic Remote-sensing Instrument for Stereo Mapping (PRISM) data was utilized to enhance the spatial resolution of Sentinel 2 data. PRISM is mounted on the well-known ALOS (Advanced Land Observing Satellite). PRISM was specifically utilized for digital elevation mapping with a pixel size of up to 2.5 m (Table 1) and was accessible through Alaska Satellite Facility or Japan Aerospace Exploration Agency (JAXA) Earth Observation Research Center (EORC) website. Firstly, radiometric calibration was applied to PRISM data to convert the raw digital numbers to radiance values. Then, geometric correction was performed through orthorectification and georeferencing to remove the spatial distortions. Then, we applied the Gram-Schmidt Pan Sharpening method to preserve the spectral information of the sentinel 2 bands while enhancing their spatial resolution using PRISM data. The following software was used for preprocessing and processing of the satellite images, 1- Sentinel Application Platform (SNAP), 2- ENVI v. 5.6.2. software; https://www.l3harrisgeospatial. com/Software-Technology/ENVI), which is mainly utilized for image processing, and 3- ArcGIS Desktop 10.8. (https://www.esri.com/en-us/arcgis/products/arcgis-desktop/overview/).

**Table 1:** Characteristics of Sentinel 2 and PRISM datasets.

Sentinel 2			
Band	Spectral region	Central wavelength (µm)	Spatial resolution (m)
B1	Ultra blue	0.443	60
B2	Blue	0.490	10
B3	Green	0.560	10
B4	Red	0.665	10
B5	VNIR	0.704	20
B6	VNIR	0.740	20
B7	VNIR	0.782	20
B8	VNIR	0.842	10
B8a	VNIR narrow	0.865	20
B9	SWIR water vapor	0.945	60
B10	SWIR cirrus	1.375	60
B11	SWIR	1.610	20
B12	SWIR	2.190	20

PRISM			
Number of bands	1 (Panchromatic)		
Wavelength	0.52 to 0.77 µm		
Number of optics	3 (Nadir; Forward; Backward)		
Base-to-height ratio	1.0 (between Forward and Backward view)		
Spatial resolution	2.5 m (at Nadir)		
Swath width	70 km (Nadir only)/35 km (Triplet mode)		
S/N	> 70		
MTF	> 0.2		
Number of detectors	28,000/band (Swath Width 70 km)		
	14,000/band (Swath Width 35 km)		
Pointing angle	− 1.5 to + 1.5 degrees (triplet mode, cross-track direction)		
Bit length	8 bits		

**Figure 3:**
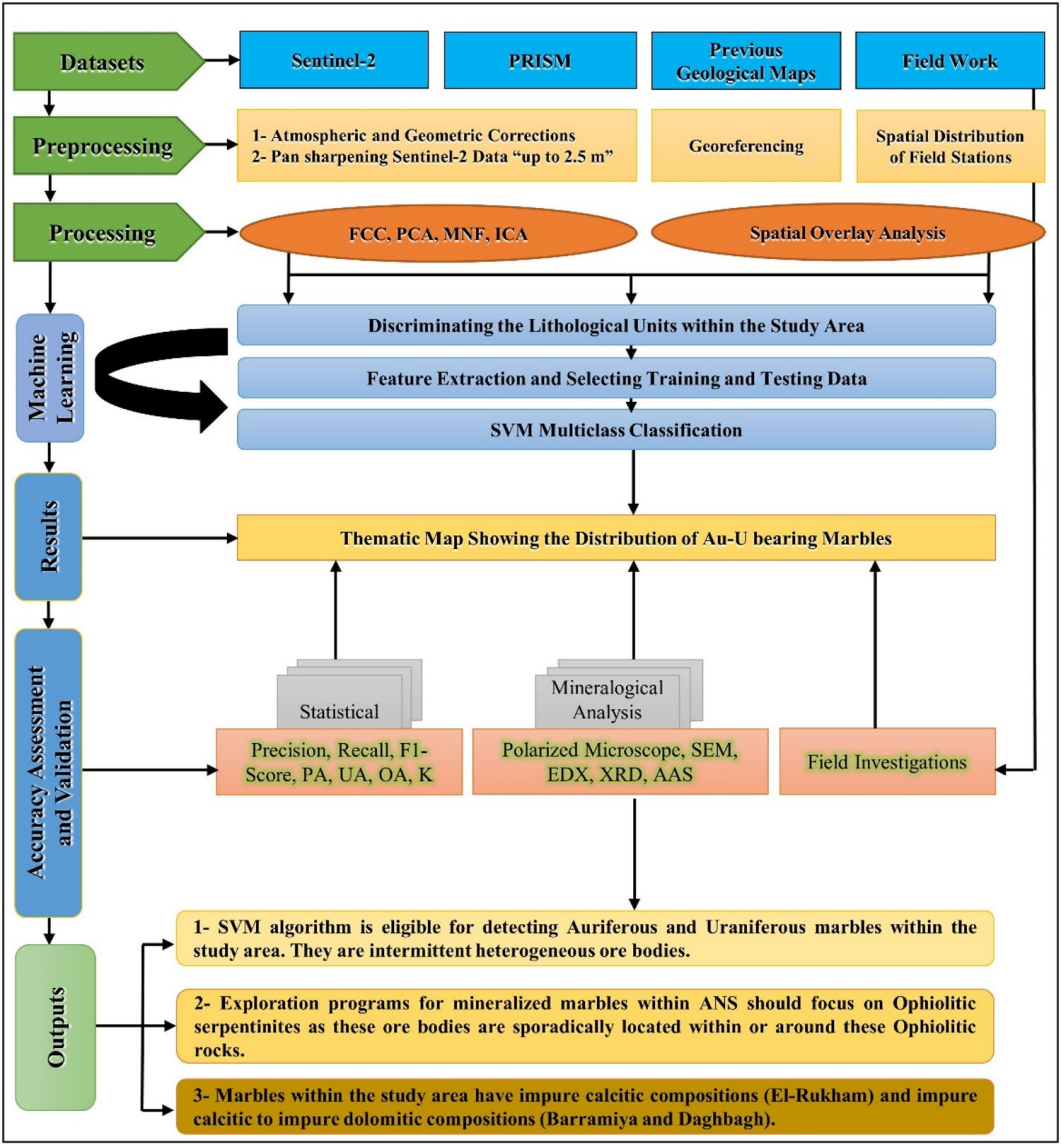
Flowchart Methodology illustrating the adopted approach in the current research.

Besides the aforementioned remote sensing datasets, previous geological maps of the study area were georeferenced and compiled from previous studies^2,39,77–79^, to allow for comparison and enhance our understanding of the study area's geology. During fieldwork, the original localities of auriferous-uraniferous marbles in Barramiya-Dghbagh district described by Hamdy and Ali^2^ were revisited and new sites identified by remote sensing and machine learning techniques were validated. A comprehensive flowchart methodology, showing the utilized datasets and elucidating the adopted approach in the present research, is introduced in Fig. 3 to provide enhanced clarification.

### Methods

#### Image processing techniques

Several image processing methods were utilized in the current research for separating serpentinites and their related auriferous-uraniferous marbles from the other lithologies exposed within the study Barramiya-Daghbagh district. Upon several trials using various image enhancement techniques, four methods (false color combination or FCC, principal component analysis or PCA, minimum noise fraction or MNF, and independent component analysis or ICA) proved their efficiency in delivering considerable lithological discrimination and acceptable delineation for the mineralized marbles. Although, FCC is a traditional remote sensing method, it is still widely used for various applications by specifying three bands in RGB. Selecting these bands depends mainly on the feature to be scrutinized^42,48,80,81^. For instance, in geological remote sensing, visible near-infrared (VNIR) bands are often included for discriminating iron-rich minerals due to the unique absorption features of these minerals at this spectral range. Short-wave infrared (SWIR) bands are the best choice for highlighting carbonates and OH-bearing minerals^42,82,83^. In the current research and due to the wide compositional variability of the exposed rock units, the best image composite differentiating the rock units was represented by SWIR, VNIR, and visible blue ranges through displaying Sentinel 2 band 12 (SWIR) in red, band 6 (VNIR) in green, and band 2 (blue) in blue channels. This FCC (12–6–2 in RGB respectively) (Fig. 1b), differentiates marbles from their country rocks (mainly serpentinites) especially when pansharpend with ALOS PRISM data (e.g. Figs. 8b, 9b, and 10b). Moreover, and aiming for better discrimination, image transformations were applied using principal component analysis (PCA), minimum noise fraction (MNF) and independent component analysis (ICA). PCA is a multivariate statistical method that transforms the original data into new components (PC)^37,84^. This transformation mostly reveals new features and introduces better discrimination, especially with the former highly informative components. MNF is another data orthogonal rotation technique. As the name suggests it tries to minimize the data noise by determining the PC from noise-whitened data. ICA is considered a blind source separation technique that tries to differentiate source and mixed signals without any previous knowledge depending mainly on defining independent uncorrelated data^49^. PCA has been performed over only the highly informative composite (12–6-2 in RGB). Additionally, MNF and ICA were applied to confirm the lithological separation and help in discriminating the mineralized marbles in the Barramiya-Daghbagh district.

#### Sampling

A crucial phase in the lithological mapping process is choosing representative samples for training and testing the model and validating the final thematic map. In the current research and based on the accessibility, 40 representative samples were acquired (before applying SVM) through intensive field investigations. The latter was performed based on previous geological maps and the results of image processing techniques (FCCs, PCA, MNF, and ICA) that deliver reasonable identification of different lithological units. These samples represent all the lithological targets within the study area and their known locations were used for picking training and testing data for the SVM model.

#### Features extraction and SVM optimization

Toward better classification results, special attention was paid to extracting the best features representing each class (lithological target). Thus, the high spatial resolution ALOS PRISM data (2.5 m), previously-mentioned image processing techniques, field observations, and previous georeferenced (to WGS 84 UTM zone 36 N) geological maps^39,77,79^ were integrated to detect the best representative pixels for six main classes including, 1- serpentinites, 2- granites, 3- metagabbro-diorites, 4- metavolcanics and volcaniclastic metasediments, 5- wadi deposits, and 6- auriferous-uraniferous marbles. According to previous studies^85^, training and testing data were kept between 70–80% and 30–20%, respectively. The data split was conducted randomly. The training and testing data of the six classes were accurately selected (Table 2) focusing on auriferous and uraniferous marbles and the surrounding rock units. These numbers of pixels (displayed in Table 2) were determined based on a combination of oversampling and undersampling trials to balance out the dataset and ensure the best representative samples for each class, based on our fieldwork and previous geological maps. A closer investigation of mineralized marbles was achieved by incorporating parts of different locations (Barramiya -BM, El-Rukham- ER, and Daghbag -DG) of confirmed auriferous-uraniferous marbles in the testing data.

**Table 2:** Characteristics of training and testing data.

Class	Training data		Testing data	
	Pixels	%	Pixels	%
SP	403	75.75	129	24.24
GR	443	78.26	123	21.73
MVs	427	76.93	128	23.06
GB	408	72.34	156	27.65
WDs	407	74.81	137	25.18
MB	252	70.19	107	29.80

Serpentinite (SP), Granitic rocks (GR), Metavolcanics and volcaniclastic metasediments (MVs), Gabbroic rocks (GB), Wadi deposits (WDs), and Au-U-bearing marbles (MB).

Towards a more balanced classification, updating the lithological map of the study area, and unraveling the spatial relationship of Au and U-bearing zones with the surrounding rock units, a multiclass classification was performed by feeding the SVM algorithm with Sentinel 2 data to classify the study area into the six main classes. SVM is picked out as it is considered one of the best classifiers in performing remote sensing data multiclass generalization^55,78,86^ and depends on statistical learning theory^87^. SVM is based mainly on achieving the maximum separation among the classes using an optimal hyperplane. What makes its efficiency much better is that, besides this margin, a misclassification penalty is always applied helping in better classification. With reference to similar previous studies^44,55^ and after several trials, the optimal parameters for SVM were a radial basis function (better than linear, and polynomial) as a kernel, and 100 for the penalty. According to our several trials and similar prior studies^44,49,51^, the reciprocal of the input bands was recommended and subsequently used to assign the value of 0.33 to the gamma parameter within the kernel function.

It should be emphasized that manually selecting the optimal hyperparameters through trial and error is a cumbersome task. Therefore, we conducted more than 50 classification trials to achieve the best fitting (as overfitting and underfitting issues are related mostly to the selected parameters) and ensure that the rock units in our study area were allocated appropriately. Besides the trial and error approach, we consulted several previous studies^44,49,51^ that yielded favorable results in similar terrains and conditions for picking the optimal parameters assigned in our research.

#### Microscopic and mineralogical analysis

Representative samples from the new sites of the auriferous-uraniferous marbles from the Barramiya-Dghbagh district were investigated microscopically and mineralogically at the Nuclear Materials Authority-Cairo. Samples were examined using both polarized and scanning electron microscope (SEM) for petrographic details. The SEM imaging was used to demonstrate the geometrical relationships between the mineral constituents, particularly dolomite and calcite, as well as to detect non-carbonate grains that were not visible under the polarised microscope due to their small size. The SEM is equipped with Link Analytical AN-1000/855 energy dispersive X-ray spectrometer calibrated using natural standards to identify elements and detect, semi-quantitatively, their chemical compositions. 25–30 kV of accelerating voltage were applied during the energy dispersive X-ray spectrometer analysis (EDXA). For elements with Z > 9, the analytical precisions range from 2 to 5%, and for lighter elements, they range between 5 and 10%. Using X-ray diffraction (XRD) spectroscopy, the identification of minerals and their relative abundances were verified. The concentration of Au in nine representative mineralized marble samples was detected by atomic absorption spectrophotometer (AAS). Aqua regia was used to digest samples for Au analysis. The analytical precision is ± 5%. After HCl-digestion contents of U_chemical_ and Th_chemical_ were determined spectrophotometrically (Colormetric method). Because U is not a gamma emitter, gamma-ray spectrometric determination of equivalent U (eU) is based on the measurement of gamma-rays emitted by its daughters.

## Results and discussion

### Image processing results

**Figure 4:**
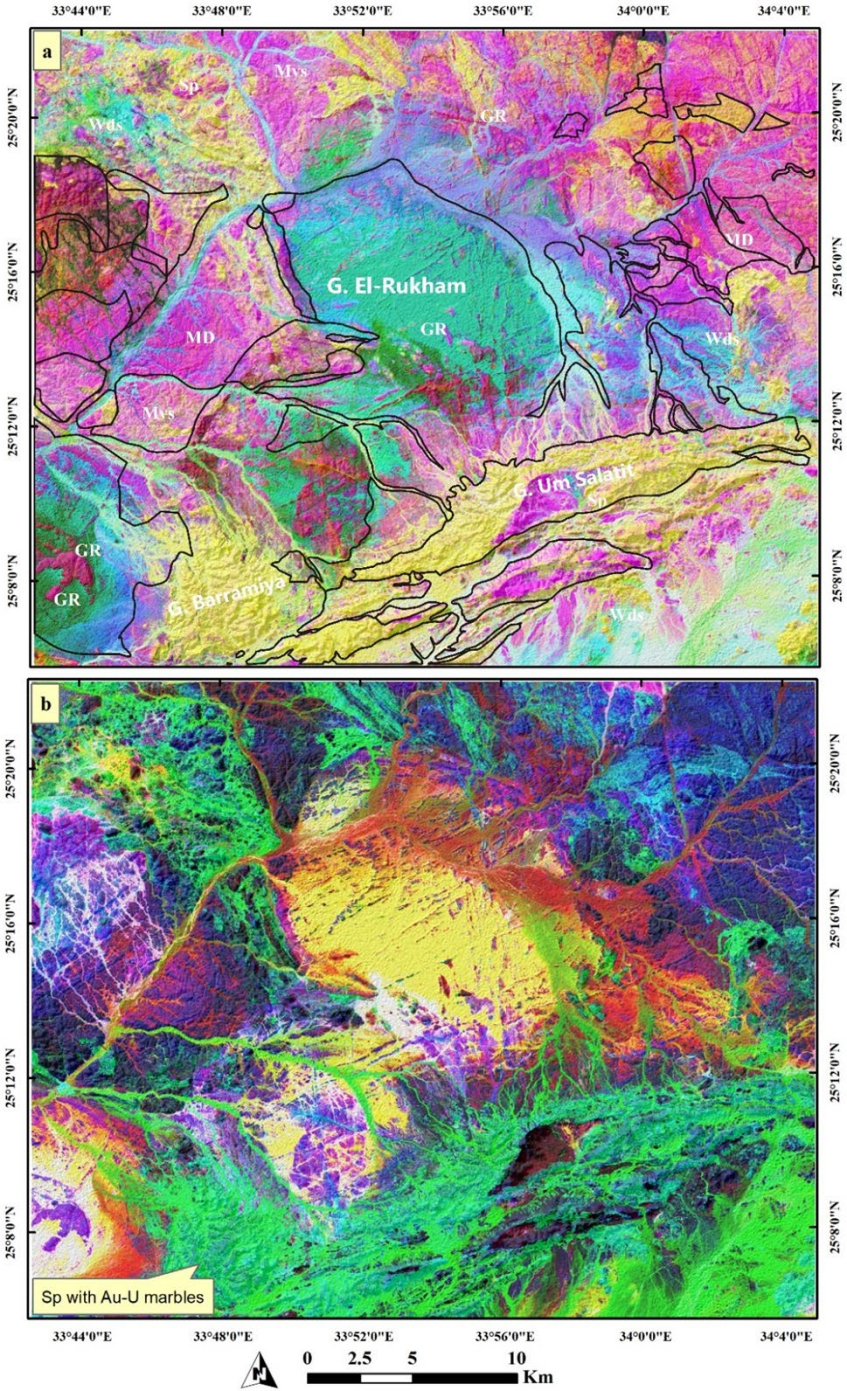
Color combinations of RGB showing (**a**) PC1-PC2-PC3 separating serpentinite rocks in yellow color with some minute pinkish impurities within it, and (**b**) MNF1-MNF2-MNF3 discriminating serpentinites and their related Au-U marbles. (Created by ENVI v. 5.6.2. software; https://www.l3harrisgeospatial.com/Software-Technology/ENVI).

**Figure 5:**
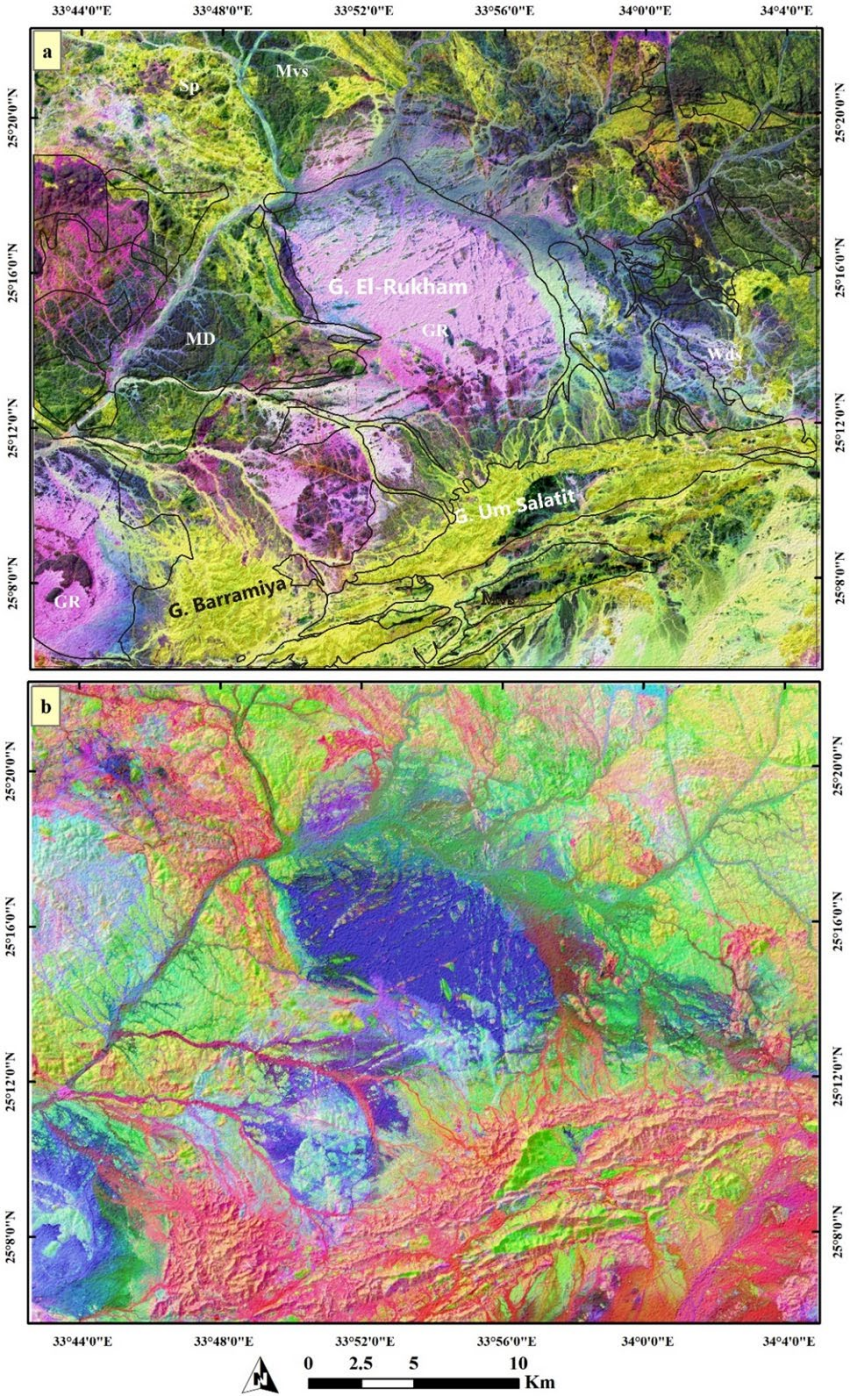
Combinations of RGB showing (**a**) MNF2-PC2-12 discriminating serpentinite rocks in yellow color with some minute greenish impurities within it, and (**b**) IC1-IC2-IC3 discriminating serpentinites in different pinkish tones. (Created by ENVI v. 5.6.2. software; https://www.l3harrisgeospatial.com/Software-Technology/ENVI).

Image processing results give a clear lithological discrimination for all the rock units within the Barramiya-Dghbagh district as shown in Figs. 4, and 5. For instance, Fig. 4a clearly separates serpentinites in yellow-colored pixels using PC1-PC2-PC3 in RGB respectively from granitic rocks (green), metavolcanics and volcaniclastic metasediments (dark pink), and metagabbros (light pink). Field observations support, to a great extent, these results and revealed that the mineralized marbles are entirely spatially-associated with the ophiolitic serpentinites. Thus, special emphasis was given to discriminating serpentinites and intensively investigating their minute varieties. Accordingly, MNF composite RGB 1–2-3 results provide better discrimination for serpentinites and their related auriferous-uraniferous marbles in two grades of green color. By checking these green colors with our field observations all over the study area, a considerable coincidence among them and the distribution of auriferous-uraniferous marbles was noticed. Of course, the green color spatial distribution is larger than the real occurrences of marbles as serpentinites and their related components (e.g. talc-carbonates) are highlighted as well, with the same color. These variations within serpentinite rocks are also confirmed by Fig. 5a (yellow color with greenish hue for serpentinites) and Fig. 5b (different tones of the pinkish color). These variations (within and around serpentinites over different parts of the study area) are confirmed utilizing ALOS PRISM data. These tonal differences within serpentinites are mainly indications for auriferous-uraniferous marbles, which have a heterogeneous composition (based on field observations) as noted from different tones within serpentinite rocks (clearly seen in the southern and northern parts of the study area and in Figures of 8b, 9b, and 10b of PRISM images).

### SVM thematic map

**Figure 6:**
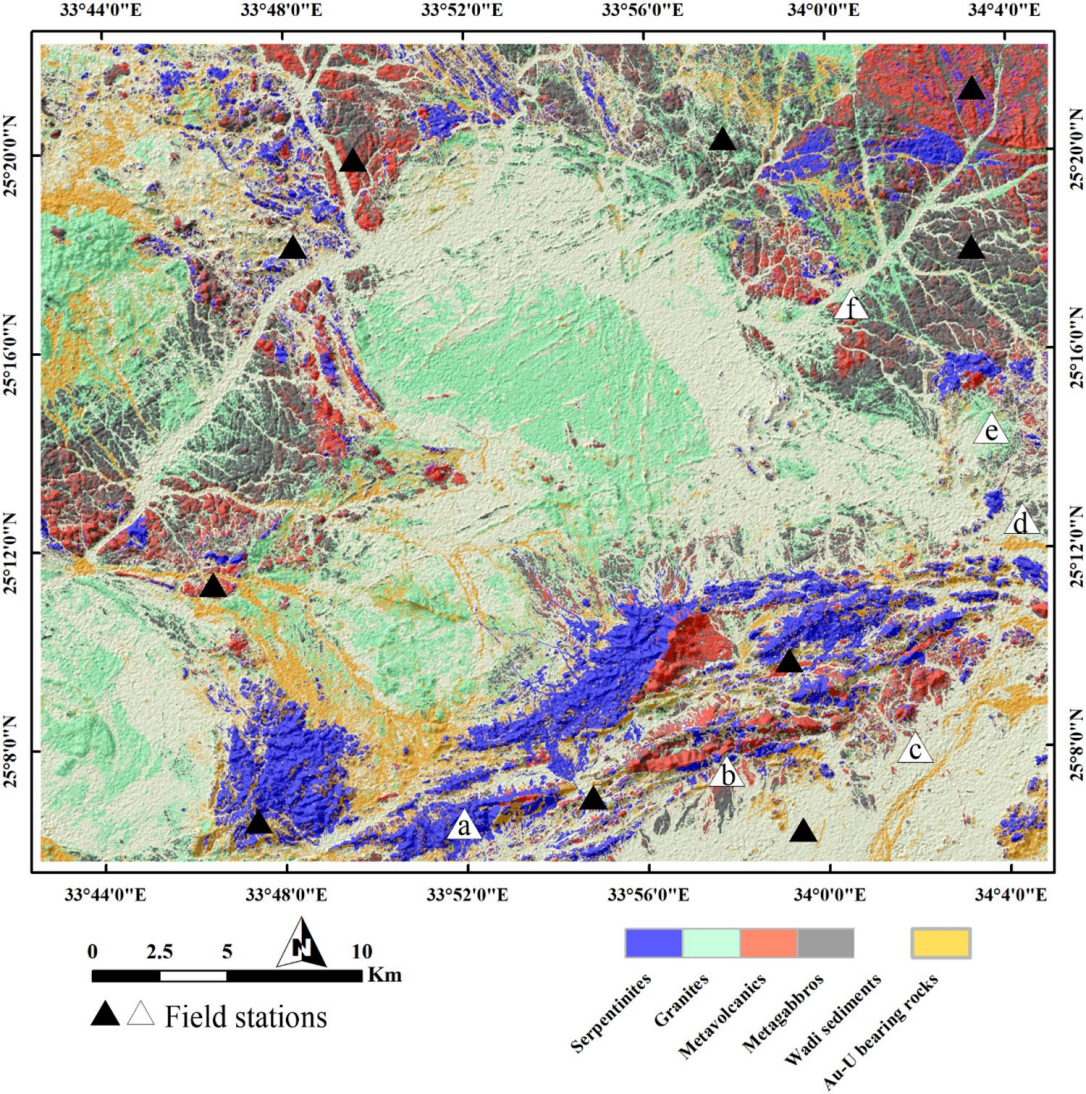
Thematic lithological map of the study area created using SVM and showing the distribution of Au-U-bearing marbles (yellow) within the study area. Created by ArcGIS Desktop 10.8. https://www.esri.com/en-us/arcgis/products/arcgis-desktop/overview and ENVI v. 5.6.2. software; https://www.l3harrisgeospatial.com/Software-Technology/ENVI.

**Figure 7:**
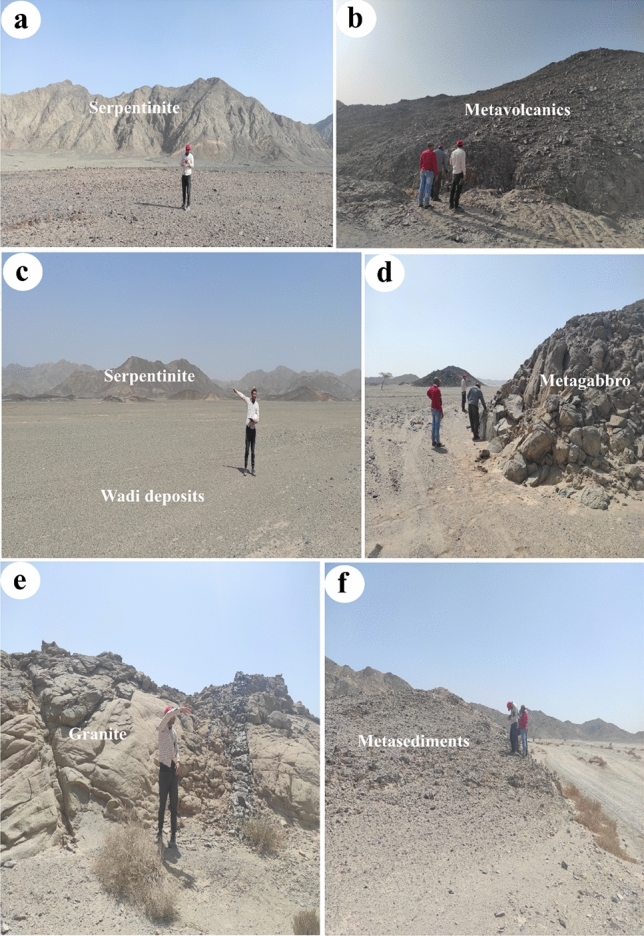
Field photographs validating the main classified rock units including (**a**) serpentinites of G. Um Salim, (**b**) metavolcanics, (**c**) Um Salatit serpentinite and Wadi deposits, (**d**) Metagabbro, (**e**) granitic rocks, and (**d**) volcaniclastic metasediments introduced through the final SVM thematic map. The exact locations of these field photographs are dropped over Fig. 5 inside white the triangles. These photos are our own and we agreed to publish them.

**Figure 8:**
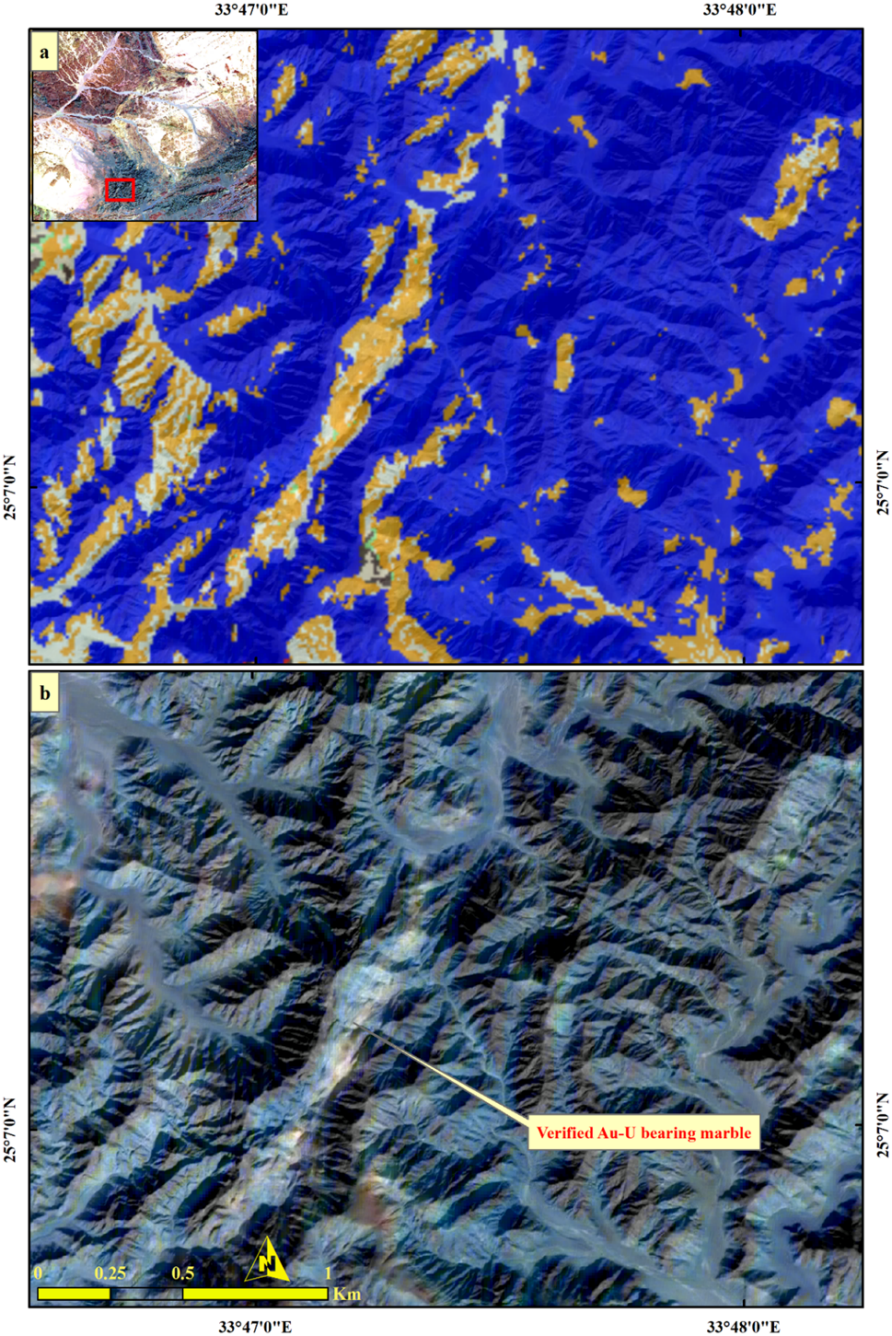
Validating (**a**) SVM result of Au-U-bearing marbles through comparison with (**b**) a well-known mineralized banded zone within the Barramiya area using PRISM Pan-sharpened (2.5 m) Sentinel 2 12–6-2-FCC in RGB. Sentinel 2A image was downloaded through the European Space Agency (ESA) platform. PRISM data was accessible through Alaska Satellite Facility and Japan Aerospace Exploration Agency (JAXA) Earth Observation Research Center (EORC) website. The figure was created by Sentinel Application Platform (SNAP), 2- ENVI v. 5.6.2. software; https://www.l3harrisgeospatial. com/Software-Technology/ENVI), which is mainly utilized for image processing, and 3- ArcGIS Desktop 10.8. (https://www.esri.com/en-us/arcgis/products/arcgis-desktop/overview/.

**Figure 9:**
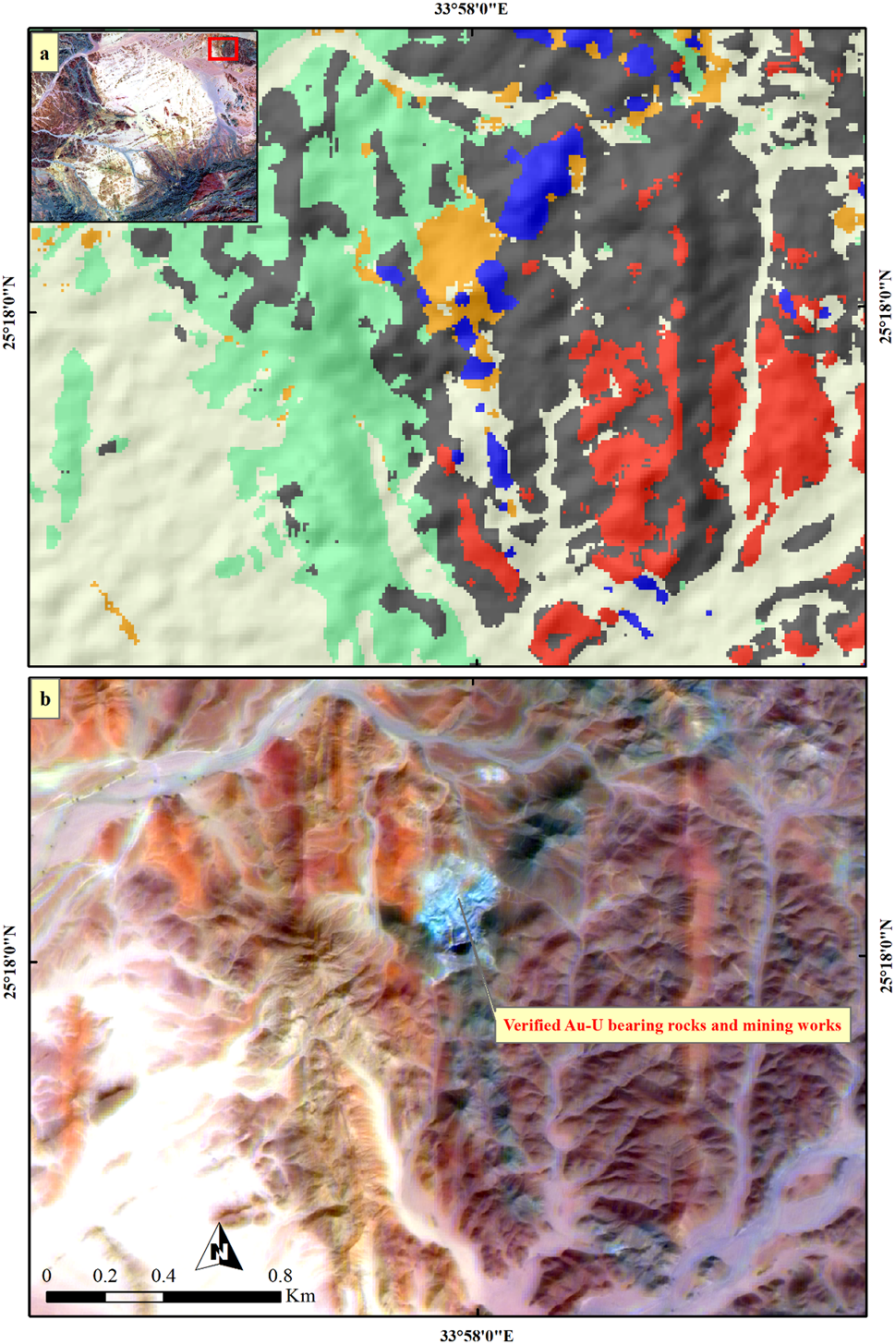
Validating (**a**) SVM result of Au-U-bearing marbles through comparison with (**b**) a well-known mineralized zone at El-Rukham area using PRISM Pan-sharpened (2.5 m) Sentinel 2 12–6-2-FCC in RGB. Sentinel 2A image was downloaded through the European Space Agency (ESA) platform. PRISM data was accessible through Alaska Satellite Facility and Japan Aerospace Exploration Agency (JAXA) Earth Observation Research Center (EORC) website. The figure was created by Sentinel Application Platform (SNAP), 2- ENVI v. 5.6.2. software; https://www.l3harrisgeospatial.com/Software-Technology/ENVI), which is mainly utilized for image processing, and 3- ArcGIS Desktop 10.8. (https://www.esri.com/en-us/arcgis/products/arcgis-desktop/overview/.

**Figure 10:**
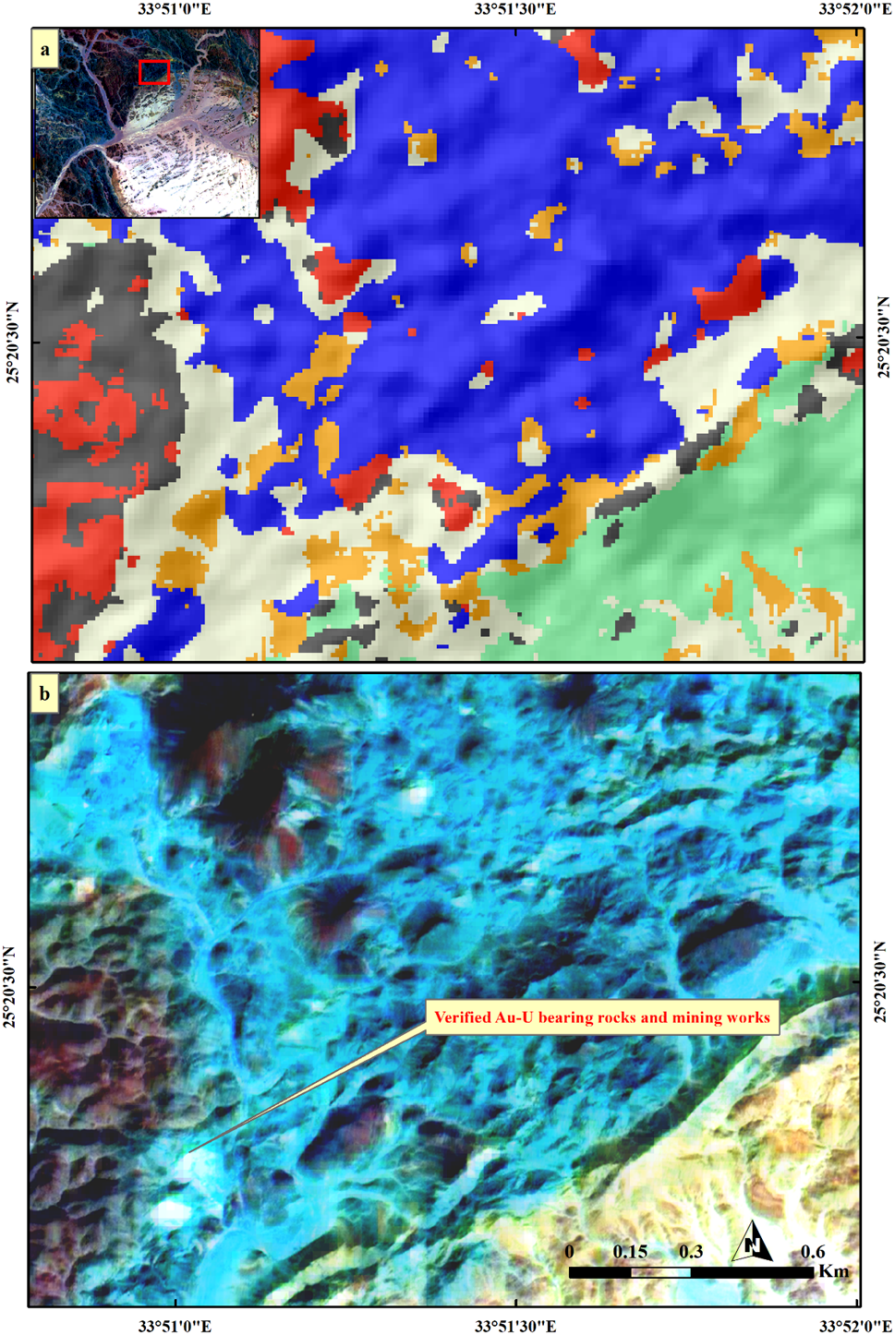
Validating (**a**) SVM result of Au-U-bearing marbles through comparison with (**b**) a well-known mineralized zone at Daghbagh area using PRISM Pan-sharpened (2.5 m) Sentinel 2 12–6-2-FCC in RGB. Sentinel 2A image was downloaded through the European Space Agency (ESA) platform. PRISM data was accessible through Alaska Satellite Facility and Japan Aerospace Exploration Agency (JAXA) Earth Observation Research Center (EORC) website. The figure was created by Sentinel Application Platform (SNAP), 2- ENVI v. 5.6.2. software; https://www.l3harrisgeospatial.com/Software-Technology/ENVI), which is mainly utilized for image processing, and 3- ArcGIS Desktop 10.8. (https://www.esri.com/en-us/arcgis/products/arcgis-desktop/overview/.

As an objective method to detect the distribution of auriferous-uraniferous marbles, SVM delivers a thematic map for the six classes (Fig. 6) utilized in the classification process. The overall accuracy (OA) was about 90.76% indicating a good differentiation for the classified lithological targets. Besides the OA, the resultant thematic map was assessed utilizing the well-known kappa coefficient (K), confusion matrix, and producer and user accuracies, and F1-score (Table 3), and field observations (Fig. 7). This statistical validation indicates that serpentinite rocks were clearly separated from other granitic, metagabbroic, and metavolcanic rocks. Generally, all the producer accuracies were above 90% for all the classes. Misclassifications are mainly the result of complicated spectral characteristics and varied topography that could result in sun illumination issues in image classification. For instance, in Figs. 8, 9, and 10, the scale of observation is large (in meters), that's why minute details of shading issues could be depicted. Nevertheless, our primary focus was on the classification of mineralized marbles, which exhibited fewer instances of these issues in the classified images compared to other rock units.

**Table 3:** Confusion matrix, Overall accuracy (OA), Kappa coefficient (K), Recall and Producer Accuracy (PA), and Precision and User Accuracy (UA) of SVM classification results.

S2	SP	GR	MVs	GB	WDs	MB	Tot	PA (%)	UA (%)	F1 Score (%)
SP	129	0	0	0	0	0	129	100	100	100
GR	0	103	0	3	0	10	116	83.74	88.79	86.10
MVs	0	0	127	0	0	0	127	99.22	100	99.61
GB	0	0	1	153	0	0	154	98.08	99.35	98.71
WDs	0	19	0	0	130	31	180	94.89	72.22	81.34
MB	0	1	0	0	7	66	74	61.68	89.19	72.30
Tot	129	123	128	156	137	107	780	OA = 90.76 K = 0.88		

Serpentinite (SP), Granitic rocks (GR), Metavolcanics and volcaniclastic metasediments (MVs), Gabbroic rocks (GB), Wadi deposits (WDs), and Au-U-bearing marbles (MB).

A field check of the main lithological units within the study area revealed a reasonable match between the resultant thematic map and our field observations (Fig. 7) however, some misclassifications are almost evident among granitic rocks, wadi deposits, and auriferous-uraniferous marbles. For instance, most of the granitic rocks within the study are syn-tectonic granites which are highly dissected, fractured, and weathered as shown around the G. El-Rukham area. Additionally, the mineralized marbles are heterogeneous rocks (black and white marbles) with a chemical favourability to rain water during storms that occasionally affect the study area. Thus, a considerable amount of serpentinite products and maybe marbles are almost seen around serpentinites and along the surrounding wadi deposits. These wadi deposits have a higher potentiality of gold placer deposits compared to others within the study area. This is confirmed by the abundance of random mining within the study area in the altered serpentinites and their placers.

These findings are confirmed by the detailed statistical analysis of our target class (MB) through calculating and interpreting their recall, precision, and F1-score. According to the classification results, the SVM model looks to have a decent accuracy of 89% for categorizing mineralized marbles. This indicates that the SVM model is 89% of the time right when it predicts that a certain pixel is a marble. This shows that, out of all samples it predicts as positive, the model is able to properly identify a significant portion of positive samples (i.e., samples corresponding to mineralized marbles). This results in less false positives (i.e. situations when the SVM model predicts that a certain pixel is mineralized but it is not). In exploration programs where false positives might have repercussions since they may prompt pointless or expensive exploration efforts, this situation is always favored. Therefore, it is important to minimize false positives when exploring mineralized rocks.In the current classification, the given precision is about 89% and the error percentage is identified in the resultant thematic maps, where a considered number of pixels (representing wadi deposits) are misclassified as auriferous-uraniferous marbles. However, the low recall of 61.68% indicates that the SVM model is missing a significant number of mineralized marbles. In the current study, this is attributed to variability in the appearance of mineralized marbles in satellite images which is confirmed during fieldwork (black and white marbles could be present within the study area) besides confusions with wadi deposits. This could also be attributed to the complexity of the classified targets where the spectral signatures of the rock units are barely differentiated, affected by several tectonic events, hydrothermal alterations, and weathering processes. Whether or not this level of performance is "good" depends on the classification context and the acceptable trade-off between precision and recall. In some cases (e.g. the current study), high precision may be more important than recall, as, if false positives (i.e., non-mineralized rock units being classified as mineralized) are particularly costly. In this study, missing a number of mineralized pixels might not be problematic since such areas could be further identified during the detailed field exploration programs through structural analysis or lithological relationships. As we expect that the missed pixels are more or less closer to the identified ones besides serpentinite rocks. Thus, it is crucial to emphasize that in classifications of this nature, careful consideration should be given to the trade-off between minimizing false positives and false negatives. This is essential to avoid unnecessary exploration activities or overlooking potential mineralizations.

An F1-score of 72% means that the SVM model is able to achieve both high precision and recall, although not at the same time. This suggests that the model is making a reasonable trade-off between the two measures. Specifically, the SVM model is able to correctly identify mineralized marbles with high precision, while also capturing a reasonable number of them, as indicated by the recall value. This is in our opinion the core benefit of MLAs, i.e., solving such complicated problems to deliver an efficient thematic map that is mostly similar to the reference geological map, which takes a lot of time and effort to be established. Additionally, it highlights one of the mineralized rocks that are rarely tackled (auriferous-uraniferous marbles) due to its lack of detection.

**Figure 11:**
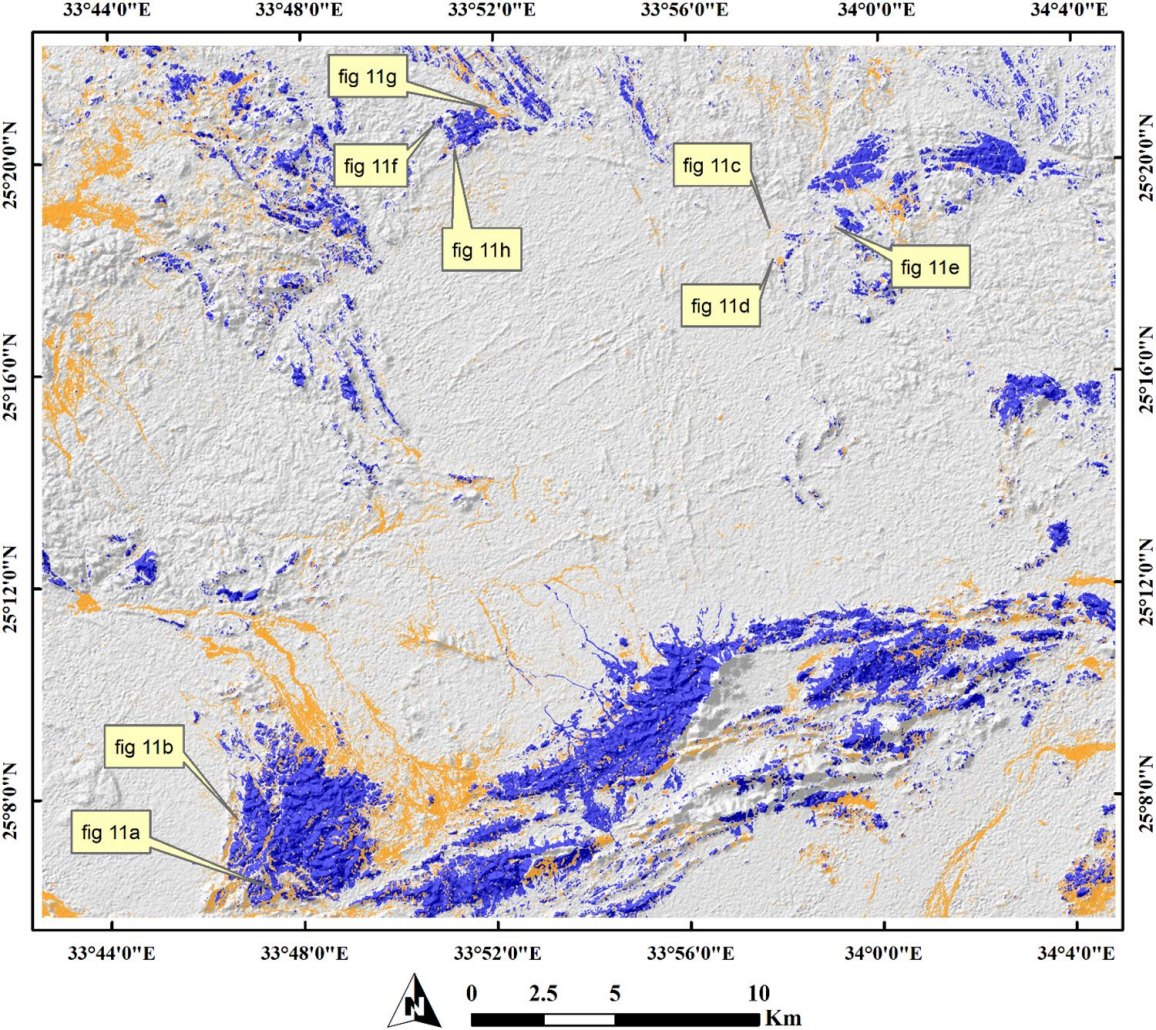
Distribution of Au-U-bearing marbles (orange) within the study area. Spatial overlay of serpentinites (blue) and Au-U-bearing marbles (orange) indicating that the latter are spatially related to (within or around) the former. Annotations (i.e. **a**) over the figure refer to the exact locations of our field observations (displayed in this figure) for Au-U-bearing marbles. A great coincidence between the SVM result and our field observations is seen where all the callout annotations point to orange pixels. Concentrations (in ppm) of Au and U in marble rocks at these locations are given in supplementary Table 1. Created by ArcGIS Desktop 10.8. https://www.esri.com/en-us/arcgis/products/arcgis-desktop/overview and ENVI v. 5.6.2. software; https://www.l3harrisgeospatial.com/Software-Technology/ENVI.

For more verification of SVM output, three confirmed sites of auriferous-uraniferous marbles including BM (Fig. 8), AR (Fig. 9), and DG (Fig. 10) were checked within the resultant thematic map and a great coincidence (separating the mineralized marbles from the country rocks) was noticed. It is worth noting that, we took into account only two sites during the training of the SVM model. As a form of performance verification, the third site was deliberately excluded from the classifier's training data. Furthermore, it is important to note that not all pixels within the selected two sites were delineated as training data. This approach was adopted to ensure a kind of internal validation for these sites. Additionally, the spatial distribution of the mineralized marbles and serpentinites was checked (Fig. 11) within the resultant thematic map manifesting the spatial association among the mineralized marble and serpentinite rocks as confirmed during fieldwork. The predicted auriferous-uraniferous marbles were confirmed by field observations (exact locations of our field stations are dropped over Fig. 11) which is discussed in more detail in the following section.

### Field verification of auriferous-uraniferous marbles

Several auriferous-uraniferous marble occurrences in the studied Barramiya-Daghbagh district are mainly associated with the segmented thrust-bound ophiolitic mélange rocks and island-arc metavolcanics-metasediments. These marbles occur mainly at Wadi Al Barramiya, Gebel El-Rukham, and Wadi Daghbagh. Generally, all studied marbles do not exhibit stratification or schistosity, which may indicate that the original carbonate textures have been obliterated^88^. In washed crushed samples using the hand lens all marbles are fossil-free and show different grain sizes and shape of the forming carbonate minerals.

**Figure 12:**
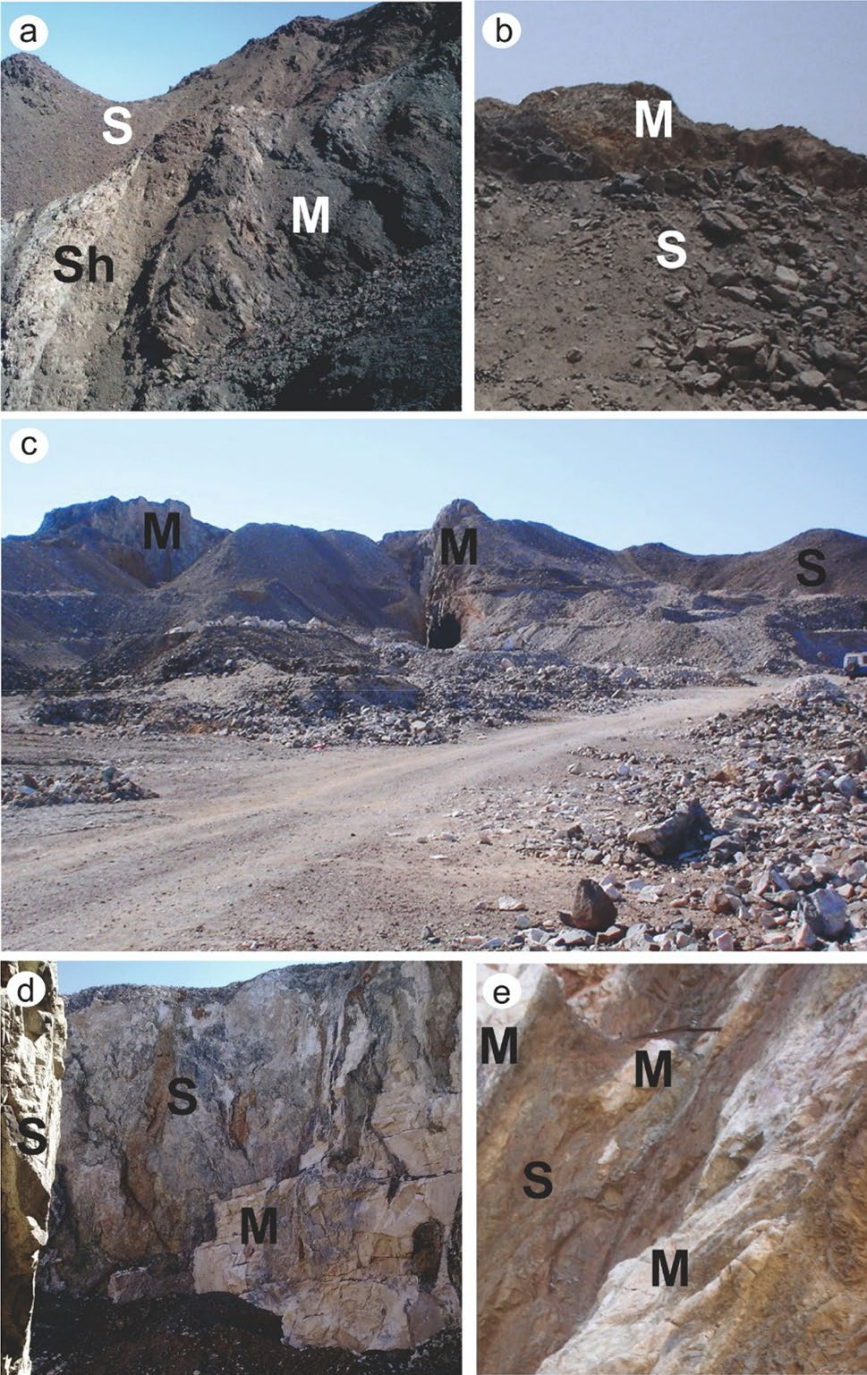

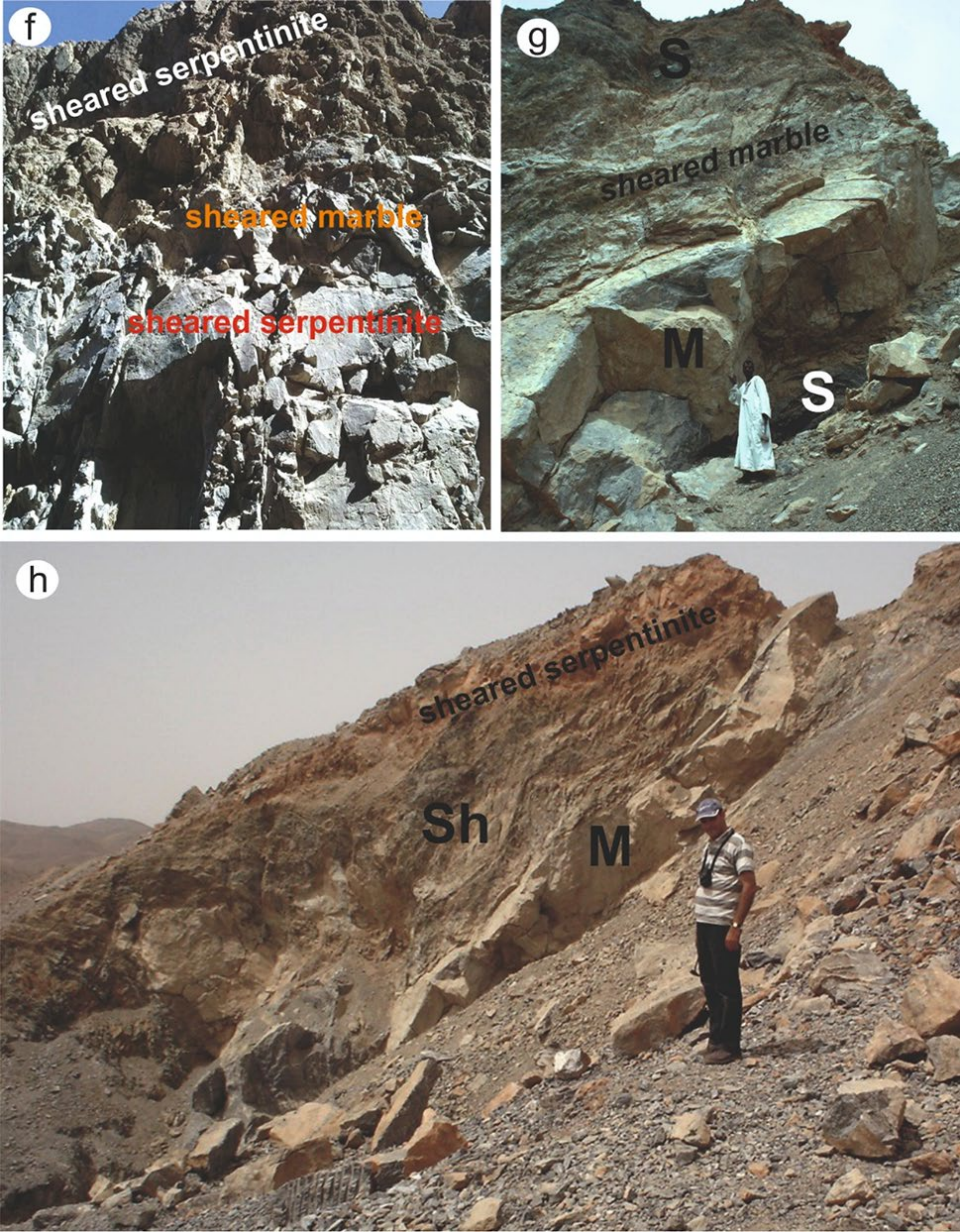
Field photographs of the auriferous and uraniferous marble rocks from (**a**,**b**) BM, (**c**,**d**,**e**) ER and (**f**,**g**,**h**) DG. S: serpentinite, M: marble, Sh: schist. Exact locations of these field photographs are dropped over Fig. 10. (These field photographs are taken by the authors of the current research. These photos are our own and we agreed to publish them.). These field photographs are taken by the authors of the current research. These photos are our own and we agreed to publish them.

In general, Barramiya-Daghbagh marbles are occasionally deformed, especially when they come into contact with country rocks (Fig. 12a). Gold mineralization can be found in both deformed and massive marble. Mineralized marble occurs abundantly in the Wadi Al Barramiya area of the Barramiya-Daghbagh district, where it is interlayered with serpentinites and sometimes schist. The occurrence of marble in the south-central part of Wadi Al Barramiya area is illustrated in Fig. 12a. Coinciding with remote sensing results (Fig. 8), the BM-marble is usually found in pod-like and bedded shapes (5–8 m thick and up to 100 m long) striking to NE-SW. It is typically gray to grayish-white. Their country serpentinites rocks are mostly altered. The contact between marble and altered serpentinite is usually not sharp. At the contact with marble, typical outcrops expose the serpentinites as highly sheared, foliated, and sometimes folded, and become rich in carbonates, graphite, and chlorite. The transition from serpentinite to marble has been described as progressive deformation of serpentinites and mylonitic marble. Extremely small particles have occasionally been produced as a result of fragmentation. Mylonitization of marble is observed at a thin section scale (2 cm). The non-mylonitic marble is dominated by equant, coarse carbonate grains. There is no obvious dimensional preferred orientation, and only a weak color banding (grey and white) defines the foliation in hand specimen.

The ER-marble occurs in slices and pod-like shapes from the cm-scale to the m-scale (up to 5 m thick) interbedding with the serpentinites and gabbros (Fig. 12b,c), particularly in the north-eastern part of the area (as confirmed by Fig. 9 from remote sensing data). They strike WNW to ESE along high-angle faults up to 60 m long in highly deformed and altered rocks (Fig. 12). Marbles are usually pure in white and coarse-grained. However, at the contact with the host rocks, they gain brownish and reddish hues. Recrystallization products are widespread within the contact aureoles with the host rocks, where marbles become zoned, with rougher textures. These contact aureoles are rich in silicate minerals which might be observed by the necked eye. Host rocks close to and at the contact with marble are also imparted by brownish shades, where minerals of carbonates, chlorite and altered chromite have frequently been encountered. The ER marble, unlike the BM marble, does not exhibit mylonitization even at the contact with the host rocks.

The DG-marble usually occurs in massive beds (2–7 m thick and up to 60 m long), but does not show stratification (Fig. 12 d, e), with a nearly NW–SE strike and dip about 20°. It occurs abundantly in the southern half of the area of W. Dghbagh. When compared to the BM and ER marbles, the DG-marble is fine-grained and has a darker black colour. It typically has coarse-grained calcite veinlets with late cross-cutting. Coinciding with remote sensing results at the north western part of the study area, DG-marble is commonly found with altered serpentinites, mylonitic graphite, and chlorite schist. The tectonic contacts between marble and the surrounding rocks are prevailed by the formation of interlayer detachment fractures. Small dike-like bodies of tonalite to granodiorite intrude the serpentinite in places and some quartz veinlets traverse the felsic bodies. Along thrust and shear zones the country serpentinites show high alterations with the development of a range of talc and yellowish-brown cavernous talc-carbonate rocks, and marble on the other hand becomes richer in silicate minerals.

### Petrographic and mineralogical features

**Figure 13:**
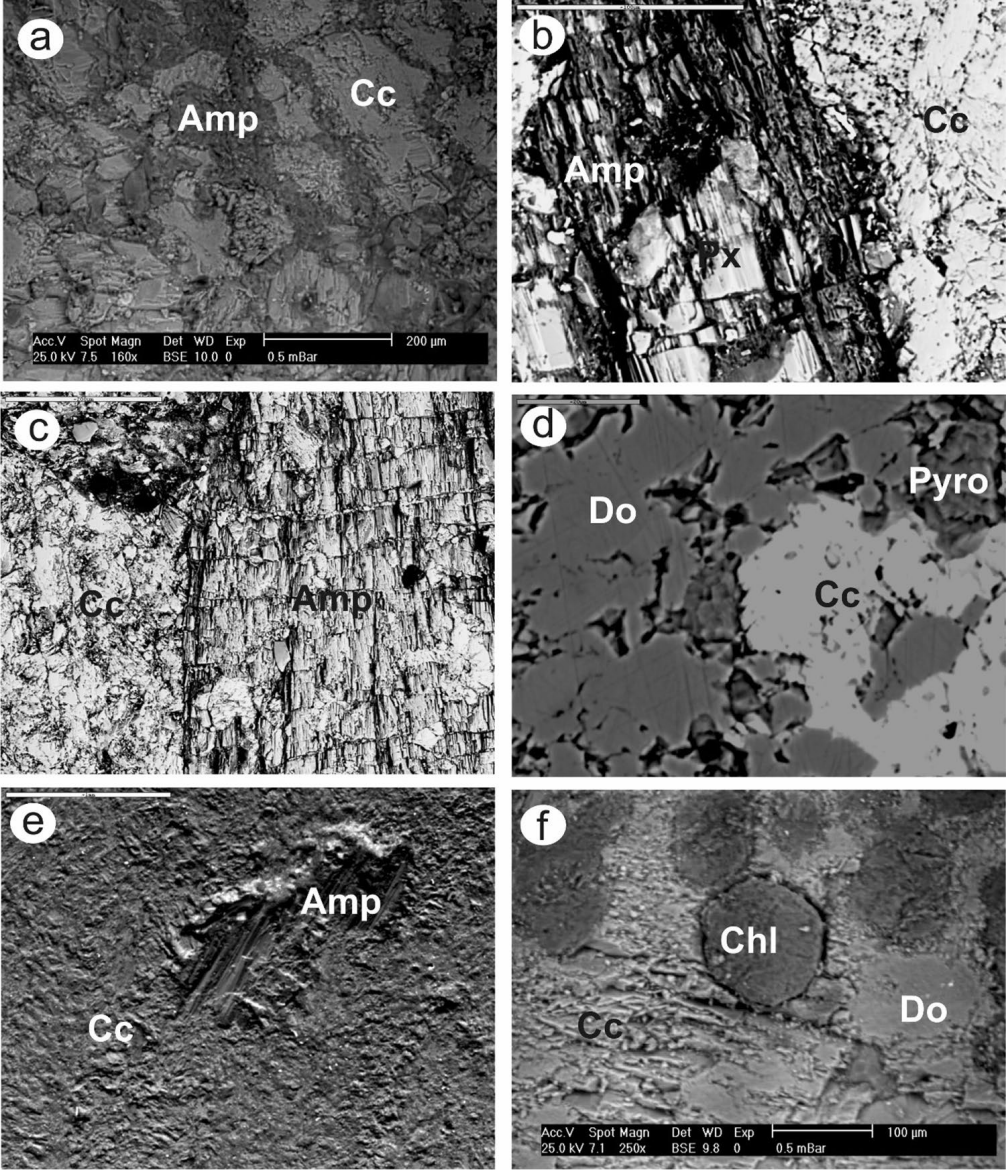

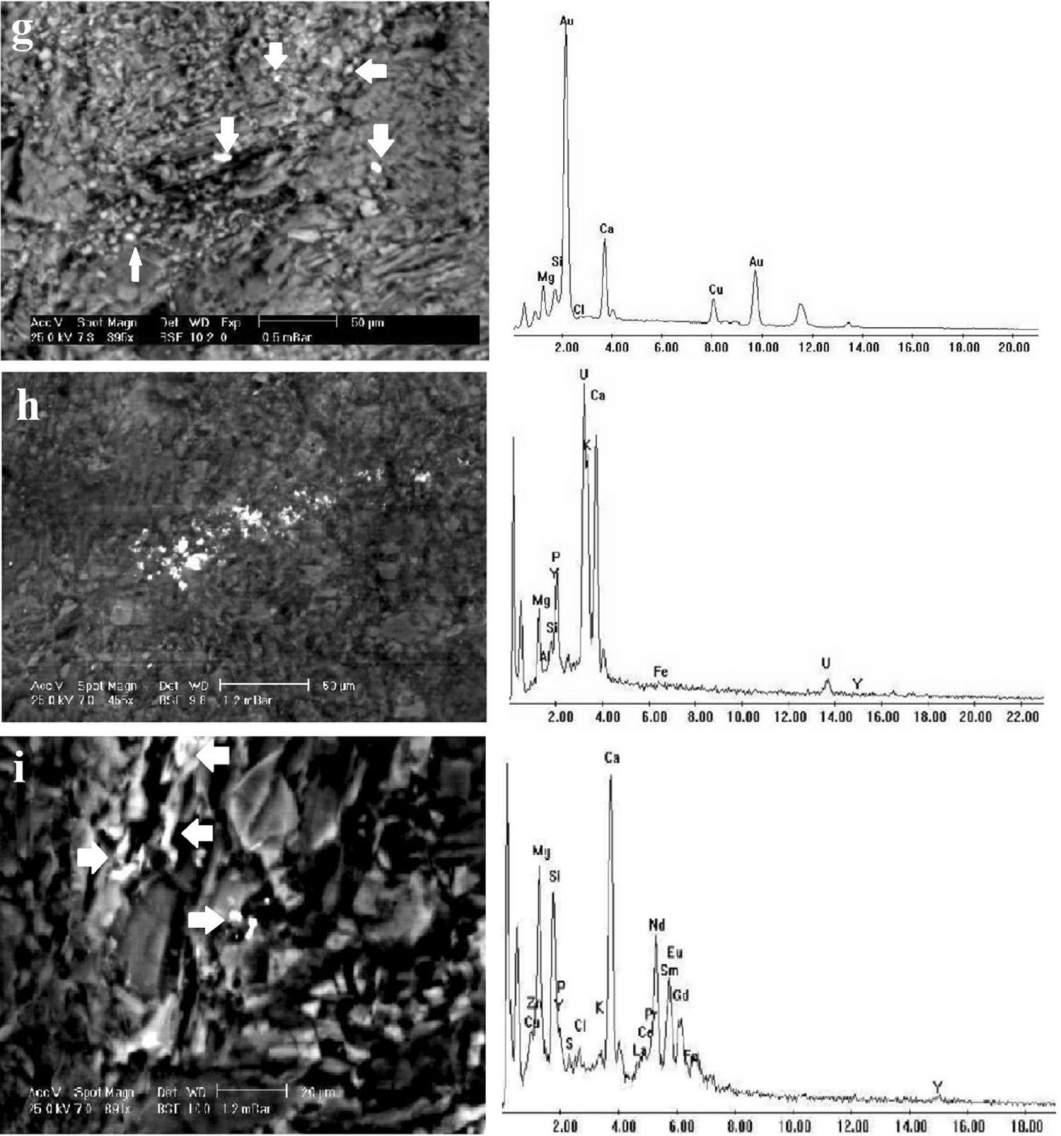
(**a**–**f**) Back-scattered electron images (BSEIs) of silicate minerals (Amp: amphibole; Px: pyroxene; Pyro: pyrophyllite; Chl: chlorite) among carbonate mineral grains (Cc: calcite; Do: dolomite), (**g**–**i**) BSEIs and EDX of gold (g), autunite (h) and REE-rich apatite (i).

**Figure 14:**
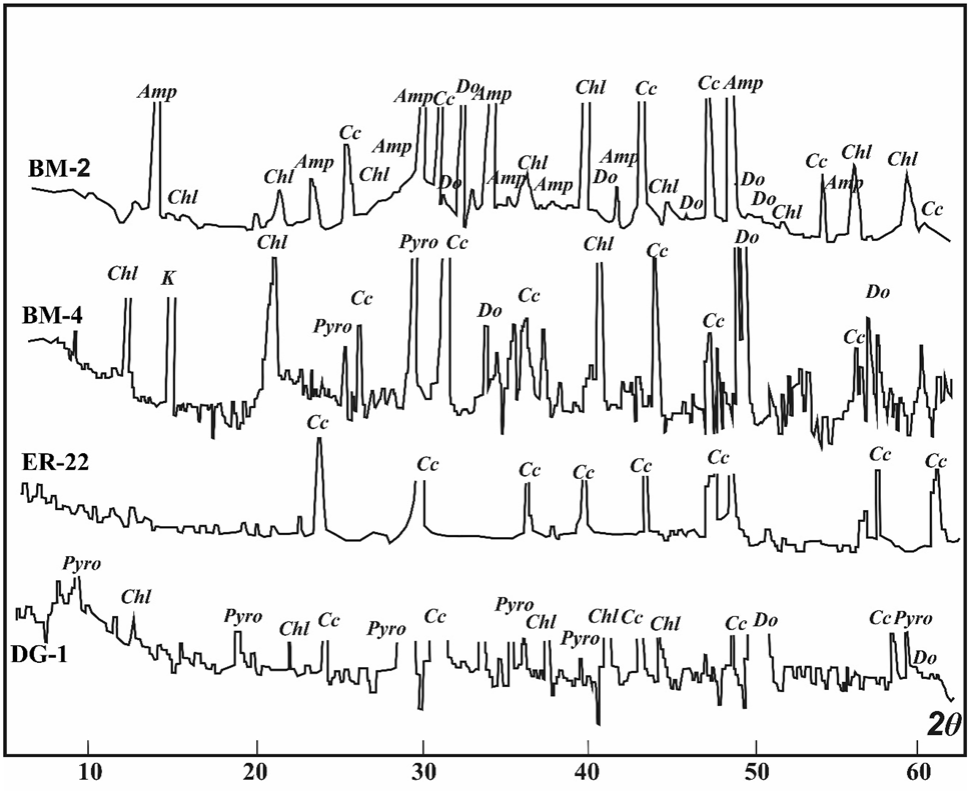
X-ray diffraction pattern of Barramiya-Daghbagh marble. Samples BM-2, BM-4 (W. Al Barramiya), ER-22 (Gabal El-Rukham), and DG-1 (W. Daghbagh). Cc (calcite), Do (dolomite), Amp (amphibole), Chl (chlorite), Pyro (pyrophyllite).

Marbles from the Barramiya-Dghbagh district contain 75–95% carbonate minerals (calcite and dolomite, except in the ER-marble it is primarily calcite) and 8–25% non-carbonate minerals, as estimated by microscopic examination supplied with EDX (Fig. 13) and XRD analysis (Fig. 14). Non-carbonate minerals found in BM-marble include amphibole and chlorite, while DG-marble contains chlorite and pyrophyllite. All samples contain, along with the gold and uranium minerals of autunite, uranophane, carnotite, and uranothorite, accessory minerals of quartz, apatite, chromite, hematite, goethite, bunsenite (NiO), danbaite [(Cu–Zn) O], REE-minerals (monazite and allanite), zircon and baddeley. Also, minor amounts of serpentine in ER samples and of graphite and pyroxene in BM and ER samples have been encountered. According to Rosen et al. ( 2004)'s classification of marbles, on the basis of their contents of carbonate and silicate minerals, the studied BM and DG auriferous-uraniferous marbles are impure calcitic to impure dolomitic; whereas those from ER are mainly impure calcitic.

Marble samples exhibit a variety of textures (the used terms are of Heinrich, 1956; Jung, 1969; Best, 1982). They are made of various-sized grains (heteroblastic), as well as granoblastic, as the carbonate grains have straight to curved borders. Unlike the BM and DG marbles, the ER marbles have stable grain boundary configurations as evidenced by plane contact surfaces of adjacent polyhedral carbonate grains (primarily calcite) and triple-grain junctions meeting at approximately 120° angles. The accessory mineral grains are euhedral to subhedral embedding in the fine carbonates (Fig. 13). Some of the marbles (especially those from DG) are texturally defined as "microgranular," composed of too fine carbonate grains (0.05–0.3 mm) to detect intergranular geometries under the microscope. In the carbonate matrix, silicate and graphite minerals are interspersed, while other accessory minerals occur in vugs and fissures.

Calcite occurs as discrete crystals interlocked with dolomite (Fig. 13). Their grain size (0.3–6 mm for calcite; 0.2–4 mm for dolomite) increases noticeably in marbles from DG to BM to ER. The EDX results demonstrate that the concentrations of major components, as well as SrO, are generally homogeneous in calcite and dolomite, but not in minor components. Calcite in the ER-marble has the highest MgO (av. 2.7 wt. %) content, and the lowest FeO (av. 0.06 wt. %) and MnO (av. 0.05 wt. %) contents, while the lowest MgO (av. 2.08 wt. %) content is detected in calcite of DG-marble, and the highest FeO (0.47 wt. %) and MnO (0.17 wt. %) contents are detected in calcite of BM-marble. Dolomite in the BM-marble has the highest FeO (av. 0.78 wt. %) and MnO (av. 0.41 wt. %) contents, while dolomite in ER-marble has the lowest FeO (av. 0.21 wt. %) and MnO (av. 0.06 wt. %) contents. According to the estimated partition coefficients of Fe (*KD*_Fe_ < 1), Mn (*KD*_Mn_ < 1), and Sr (*KD*_Sr_ > 1) between calcite and dolomite in the mineralized marbles, we conclude that the chemical equilibrium between carbonate minerals may have been achieved^90^.

Amphibole is the most prevalent accessory silicate mineral. It is found as acicular crystals around carbonates (Fig. 13a). Also, it appears as thin subhedral prisms (up to 2 mm in length) with relict clinopyroxene (Fig. 13b,c,e). The composition of amphibole (based on Leake et al. (1997)'s IMA classification) in the BM-marble ranges from tremolite to magnesiohornblende, while it is typically composed of tremolite in the ER-marble and magnesiohornblende in the DG-marble. Pyroxene is found primarily in amphibole (Fig. 13b) and occasionally between carbonate minerals. It has the composition of augite in BM-marble (av. Wo_23.5_ En_68.25_Fs_7.67_) and of diopside (av. Wo_44.04_En_55.54_Fs_0.42_) in ER-marble. Chlorite is found in trace amounts in the DG and BM marbles. It is sometimes intergrown as lamellae within amphibole or as xenoblasts within clinopyroxene, and carbonate minerals (Fig. 13f). Talc, pyrophyllite and kaolinite are recorded only in DG-marble. Talc is enclosed within or at the rims of pyroxene. Pyrophyllite occurs as individual anhedral crystals in carbonates (Fig. 13d), or in bundles within kaolinite. Graphite is common in DG and BM, occurring between carbonate grains.

Gold (10–35 µm) occurs mainly as nuggets in pores and vugs, and sometimes in fissures in the carbonate matrix (Fig. 13g). In the ER and DG-marbles, gold appears as globules or rods, but in the BM-marble, it appears as crescents or irregular streaks. The concentration of gold (Supplementary Table 1) that was determined in the rock samples ranged between 0.98 to 2.79 g/t. Copper (7.81–9.13 wt. %) is the most common trace element in gold grains. While the Ag content in gold of ER and BM-marbles is negligible, that in gold of DG-marble ranges from 7.87 to 10.03 wt. %.

Uranium minerals are most commonly found in kaolinite, hematite, and goethite. They are mainly autunite- Ca(UO_2_)_2_(PO_4_)_2_·10–12H_2_O (10–50 µm) (Fig. 13h) and uranophane- (Ca(UO_2_)_2_(SiO_3_OH)_2_·5H_2_O) (10–15 µm). However, carnotite- K_2_ (UO_2_)_2_(VO_4_)_2_·3H_2_O (30–50 µm) and uranothorite- (Th, U) SiO_4_ (3–7 µm) occur in some samples. The U contents in marble samples (Supplementary Table 1) range from 127 to 641 ppm. Uranothorite is found in most samples as fine disseminated subhedral grains or as an irregular relict in other uranium mineral grains. This suggests that uranothorite is a primary mineral from which secondary uranium minerals were formed. Zircon is detected only in BM-marble, containing a significant concentration of U. All marbles contain apatite, which is found as subhedral to anhedral grains in vugs. The spaces between the apatite grains in the DG and BM-marbles are typically filled with graphite. Apatite is distinguished by its high REE content (Fig. 13i). Monazite and allanite within the carbonate matrix are among the REE minerals in the marbles studied. Also, monazite has high concentrations of Th and U. The uranium content in the studied marbles is in a distinct disequilibrium state. The chemically analyzed uranium (U_chemical_) is 50 to 300 fold the radiometrically determined uranium (eU).

### Genetic model of the auriferous-uraniferous marble

#### Marble protolith

The studied impure marbles from the Barramiya-Dghbagh district were formed by the metamorphic recrystallization of carbonate-dominant protolith (primarily limestones, dolostones, dolomitic limestones, or carbonatites) containing minor silicate minerals. However, the inherited compositional variations of the protolith are suggested by variations in silicate mineralogy. Concentrations of SrO (0.01–0.09 wt. %) of calcite and dolomite in the studied marbles are comparable to those of sedimentary origin marble (e.g., Borra, India:^91,92^; Sol Hamed, ED-Egypt:^5^; Engabreen, Norway:^93^; Sri Lanka:^94^). suggested that the source of SiO_2_ and Al_2_O_3_ in ER and DG-marbles is most likely country serpentinite rocks, as evidenced by the strong correlation between Cr contents and these oxides. On the other hand, SiO_2_ and Al_2_O_3_ in the BM-marble may have originated from an argillaceous precursor.

#### Metamorphic evolution

The distinct mineralogy, mineral chemistry, and textural characteristics of the studied marbles provide evidence for predominated syn- to post-metamorphic fluid activities in the Barramiya-Dghbagh district. Using the EDX results, it can be determined that the anions present in volatile-bearing minerals are primarily hydroxide and carbonate, with minor chloride and fluoride. This suggests that the metamorphism fluids were likely primarily binary H_2_O-CO_2_ mixtures with low concentrations of HF and NaCl. In marbles from ER and BM, the modal abundances of the anhydrous minerals that can be produced by decarbonation reactions, like pyroxene, are low (av. 2 vol. %). This refers to fluids with low XCO_2_ equilibrium and, on the other hand, a sizable amount of aqueous fluid that was derived externally during the progression of a retrograde metamorphism.

The shape and geometric features of mineral grains in studied marbles were used to identify and demonstrate equilibration during metamorphic evolution. The larger average size and the plane contact surfaces of adjacent polyhedral carbonate grains of carbonate grains in ER-marble suggest that the ER marble exhibits more metamorphic recrystallization when compared to other marbles studied^95^. The estimated temperature of equilibration between calcite and dolomite range from 450°C in BM-marble to 650 °C in ER-marble, using *X*_*MgCO3*_ (av. = 4.76 mol.% and av. = 5.61 mol.%; respectively) in ’s calcite thermometer. Therefore, the ER-marble may represent more obliteration of the primary textural characteristics through higher-grade metamorphic process. Furthermore, the greater recrystallization of the BM-black marble compared to the DG-black marble may be responsible for the greater discoloration during prograde reactions, which is followed by greater removal of organic carbon traces^97^. This is evidenced by the BM-marble being more sparkling than the DG-marble.

The prograde reaction is evidenced by the presence of clinopyroxene as the only silicate mineral in some samples. However, its presence as the only mineral formed by prograde metamorphism reflects the simplicity of premetamorphic rocks. Based on pyroxene thermometers from^98,99^, 2 estimated that clinopyroxene formed under granulite facies metamorphism at T = 825—975 °C (augite in BM-marble), and at T = 600–900 °C (diopside in ER-marble).

Judging from the hydrous mineral assemblages, retrograde metamorphism of the marble rocks passed to the lower amphibolite and greenschist facies. The texture of the amphiboles in all marbles may indicate retrograde rehydration of clinopyroxene. When compared to tremolite, magnesiohornblende with high Al content usually replaces augite in BM-marble. This most probably was followed by the formation of tremolite (at 500–600 °C; Winter^100^). Tremolite, on the other hand, was generated in ER-marble through the hydration of diopside. In DG-marble, tremolite formation was followed by the formation of talc (400–500 °C), then pyrophyllite (300–400 °C) and finally chlorite (179–245 °C).

#### Gold–uranium mineralization

In the studied marbles, gold is found as nuggets dispersed in a carbonate matrix and is not associated with sulphide minerals. The spatial relationship of the studied mineralized marbles with serpentinite rocks indicates their potential as a source of gold. Also, the distribution of gold mineralization is not usually related to deformation zones, implying that deformation did not play the only role in fluid feeder conduits during gold mineralization.

**Figure 15:**
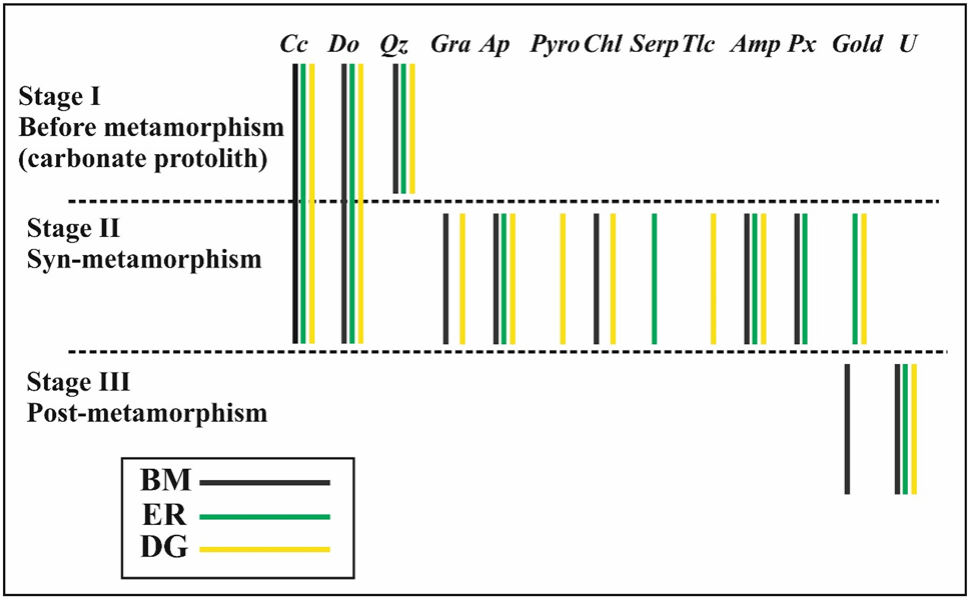
Mineral parageneses in Barramiyah-Daghbagh marble rocks.

Hamdy and Aly^2^ proposed that oxidation was responsible for the liberation of gold from the ultramafic source rocks in all marbles. In ER and DG-marbles, however, this is most likely associated with metamorphism (syn-metamorphic mineralization) and the formation of silicate minerals (Fig. 15). The ultramafic rocks experienced their metamorphism at the transitional greenschist-amphibolite facies, with brittle-ductile and brittle structures along thrusts developing^101^, providing favourable channel ways for metamorphic mineralization fluid flow. As gold was transported to the carbonate rocks in hydroxyl complexes, these fluids were essentially binary H_2_O–CO_2_ mixtures with low NaCl and HF concentrations. In contrast, gold was liberated from the source rocks in the BM-marble after metamorphism and during their alteration (post-metamorphic mineralization).

The eU/Uchemical ratio is usually less than one in all studied mineralized marbles, indicating that it was recently added (i.e., the daughters which emit gamma ray are not yet produced or at least the decay series did not reach an equilibrium state). The uranium mineralization ages in the studied marbles are < 1.5 Ma (post-metamorphic), as U reaches equilibrium at about 1.5 Ma^102^. The presence of uranium in marbles after metamorphism (Fig. 15) strongly suggests that it is of secondary origin. Furthermore, uranium's secondary origin is supported by the concentration of uranium minerals as fracture- and pore-filling minerals, as well as their mode of occurrence as phosphate, silicate, and vanadate. The felsite and trachyte dikes in ER and DG, as well as the granite rocks in BM, can be considered as potential sources of primary uranium. Meteoric water was most likely responsible for the weathering of uranium from its primary source, transportation, and deposition in marble. This most likely occurred during Egypt's pluvial periods, when the Eastern Desert was flooded by surface water^103^. Secondary U-minerals (autunite, uranophane, and carnotite) could be precipitated along fractures and open voids via evaporation, complexion with ligands, or adsorption on iron oxy-hydroxides and clay minerals. The sympathetic negative relationship between U and Au contents in BM-marble suggests that the mineralizing fluid of uranium is the same as that of gold and that mineralization occurred within the last 1.5 Ma, but at different times.

## Conclusions

For the first time over the Arabian Nubian Shield, the current research integrated Sentinel 2 and ALOS PRISM data with the well-known support vector machine algorithm for detecting auriferous and uraniferous marbles. Results of the current approach have been verified statistically (confusion matrix, overall accuracy, kappa coefficient), using intensive fieldwork, and petrographic-mineralogical investigations (XRD, EDX, and BSEIs). Our research concludes the following

1. Auriferous and uraniferous marbles are not continuous ore bodies compared to the conventional hosts for Au-U. They form intermittent heterogeneous (black or white, banded or massive,…etc.) ore bodies. Their collective representation may be of practical economic value even from the marbles or their placers; this is indicated by the abundance of random mining around the detected bodies within the study area.
2. Exploration programs for auriferous and uraniferous marbles should focus on ophiolitic serpentinites and their related rocks as in most cases these ore bodies are sporadically located within or around these ophiolitic rocks in one way or another. Accordingly, higher spatial resolution (e.g. 2.5 m) remote sensing data may reveal these mineralized varieties within these ophiolitic rocks through different techniques (e.g. PCA, ICA, and MNF and their combinations).
3. Support vector machine algorithm is eligible for detecting these mineralized marbles. The SVM resultant thematic map correlates well with the previous geological map and our field investigations.
4. Spatial overlay analysis of ophiolitic serpentinites and auriferous and uraniferous marbles thematic layer confirms their associated origin.
5. Marbles have impure calcitic compositions (ER) and impure calcitic to impure dolomitic compositions (BM and DG). Their protolith is made up of pure limestones and dolomitic limestones with possible argillaceous components (BM). Metamorphism progressed retrogradely from the granulite-amphibolite facies for the ER and BM marbles and from the amphibolite facies for the DG marble to the upper sub-greenschist facies.
6. The country ultramafic rocks are the primary source of gold, and mineralization took place both syn- (ER and DG) and post-metamorphic surficial weathering (BM). The felsic rocks in the surrounding area of marble rocks are mostly the source of uranium. The uranium was most likely transported to marble by pluvial period-related meteoric and/or underground water.
7. The current research highlighted auriferous and uraniferous marbles within the study area including Barramiya, El-Rukham, and Daghbagh, and strongly recommends further detailed exploration for the other detected zones.
8. The utilized approach is strongly recommended as a preliminary multiscale (microscopic-remote sensing) exploration model to be adopted beyond the borders of the study area for the detection of auriferous and uraniferous marbles within ANS and building its regional distribution map. This could not only give insights into the regional economic impact of the auriferous-uraniferous marbles but also it could introduce an explanation for their origins based on their distribution.

## Supplementary Information

Supplementary Tables.

## Data Availability

The datasets used and/or analyzed during the current study are available from the corresponding author upon reasonable request.
